# Dietary intake and gastrointestinal symptoms are altered in children with Autism Spectrum Disorder: the relative contribution of autism-linked traits

**DOI:** 10.1186/s12937-024-00930-8

**Published:** 2024-02-28

**Authors:** Hailin Li, Saijun Huang, Jin Jing, Hong Yu, Tingfeng Gu, Xiaoxuan Ou, Shuolin Pan, Yanna Zhu, Xi Su

**Affiliations:** 1 Department of Child Healthcare, Foshan Women and Children Hospital, Foshan, Guangdong 528000 P.R. China; 2 Research Center of Children and Adolescent Psychological and Behavioral Development, Department of Maternal and Child Health, School of Public Health, Sun Yat-sen University, Guangzhou, Guangdong 510080 P.R. China; 3 Department of Maternal and Child Health, School of Public Health, Guangdong Provincial Key Laboratory of Food, Nutrition and Health, Sun Yat-sen University, Guangzhou, Guangdong 510080 P.R. China

**Keywords:** Autism spectrum disorder, Dietary intake, Gastrointestinal symptoms, Autism-linked traits, Relative contributions

## Abstract

**Background:**

Dietary and gastrointestinal (GI) problems have been frequently reported in autism spectrum disorder (ASD). However, the relative contributions of autism-linked traits to dietary and GI problems in children with ASD are poorly understood. This study firstly compared the dietary intake and GI symptoms between children with ASD and typically developing children (TDC), and then quantified the relative contributions of autism-linked traits to dietary intake, and relative contributions of autism-linked traits and dietary intake to GI symptoms within the ASD group.

**Methods:**

A sample of 121 children with ASD and 121 age-matched TDC were eligible for this study. The dietary intake indicators included food groups intakes, food variety, and diet quality. The autism-linked traits included ASD symptom severity, restricted repetitive behaviors (RRBs), sensory profiles, mealtime behaviors, and their subtypes. Linear mixed-effects models and mixed-effects logistic regression models were used to estimate the relative contributions.

**Results:**

Children with ASD had poorer diets with fewer vegetables/fruits, less variety of food, a higher degree of inadequate/unbalanced dietary intake, and more severe constipation/total GI symptoms than age-matched TDC. Within the ASD group, compulsive behavior (a subtype of RRBs) and taste/smell sensitivity were the only traits associated with lower vegetables and fruit consumption, respectively. Self-injurious behavior (a subtype of RRBs) was the only contributing trait to less variety of food. Limited variety (a subtype of mealtime behavior problems) and ASD symptom severity were the primary and secondary contributors to inadequate dietary intake, respectively. ASD symptom severity and limited variety were the primary and secondary contributors to unbalanced dietary intake, respectively. Notably, unbalanced dietary intake was a significant independent factor associated with constipation/total GI symptoms, and autism-linked traits manifested no contributions.

**Conclusions:**

ASD symptom severity and unbalanced diets were the most important contributors to unbalanced dietary intake and GI symptoms, respectively. Our findings highlight that ASD symptom severity and unbalanced diets could provide the largest benefits for the dietary and GI problems of ASD if they were targeted for early detection and optimal treatment.

**Supplementary Information:**

The online version contains supplementary material available at 10.1186/s12937-024-00930-8.

## Introduction

Autism spectrum disorder (ASD) is a heterogeneous neurodevelopmental disorder characterized by persistent deficits in social communication and interactions and the presence of restricted, repetitive behaviors (RRBs), interests, or activities (Diagnostic and Statistical Manual of Mental Disorders, Fifth Edition, Text Revision [DSM-5-TR]; American Psychiatric Association [APA], 2022). Epidemiological studies have shown a rapid increase in the prevalence of ASD in recent years [1]. A three-level meta-analysis including 79 studies reported that the pooled ASD prevalence estimates were 0.72% (95% confidence interval [CI]: 0.61-0.85%) [2]. Autism emerges in the early developmental period, typically diagnosed in childhood, but fully manifests when the social demands exceed their limited capacities [3]. Autism has underlying cognitive features [4] and often co-exists with other psychiatric, behavioral, and physical symptoms (for example, attention deficit hyperactivity disorder [ADHD], anxiety disorders [AD], obsessive-compulsive disorder [OCD], obesity, feeding, and gastrointestinal [GI] problems) [5], and impairments in autism are severe enough to cause social, occupational, or other vital functional deficits [3].

Children with ASD are more likely than typically developing (TD) peers to experience feeding problems, with many suffering from some manifestation of food selectivity, food neophobia, food refusal, ritualized eating, rapid eating, and disruptive mealtime behaviors [6]. Deficits in social communication, RRBs, sensory sensitivities, GI problems, and oral and fine motor skill impairments may be potential mechanisms underlying feeding problems in ASD [7]. Feeding problems may lead to the underconsumption of certain foods and overconsumption of a few foods. For instance, children with ASD rejected nutrient-dense foods like legumes and dairy products [8] and consumed energy-dense, nutrient-poor foods like sweetened beverages and snack foods [9]. The presence of feeding and dietary problems has both short and long-term implications for health [10]. Short-term consequences can result in weight loss, dehydration, low energy, failure to thrive, and malnutrition [11–13]. Long-term implications include vitamin deficiencies (scurvy, beriberi, rickets, vision loss, etc.) and iron, zinc, and calcium deficiency [14].

Most published studies exploring the relationship between autism-related traits and feeding problems focused on feeding behaviors rather than food intake and diet quality [15, 16]. However, the relationship between autistic traits – autism severity and feeding behaviors differed according to evaluation tools. For instance, ASD severity was positively associated with food selectivity by parent reports via the Autism Diagnostic Interview-Revised (ADI-R) but not as assessed by clinician observation using the Autism Diagnostic Observation Scale-Calibrated Severity Scale (ADOS-CSS) [7]. Moreover, studies evaluating feeding behaviors in ASD are very heterogeneous, and they show differences in assessment criteria and methodologies, including rating scales, checklists, observations, surveys, and interviews, as well as more assessments [7, 17]. Also, the absence of unique definitions of feeding behaviors in ASD leads to inconsistent and conflicting results [6, 17]. For example, one study that used multiple measures of feeding difficulties indicated that children with more severe autism engage in more disruptive behaviors when presented with nonpreferred food, but not more food-selective behaviors than those with less severe autism [18]. These issues could potentially explain the variable relationship between ASD severity and feeding behaviors. Therefore, this study focused on food groups and diet quality as the outcome of dietary intake.

The dietary balance index (DBI), which characterizes the overall diet quality and adheres to the Chinese Dietary Guidelines (CDGs) and Chinese Food Pagoda (CFP), is made up of fundamental food groups. To date, there is a relative paucity of studies quantifying diet quality based on DBI in autism. The comparative data on specific food groups among children with ASD and typically developing children (TDC) are available, but the results are inconsistent. The case-control study reported that 32 autistic children from 4 to 8 years of age consumed more servings of fruit each day compared to 23 TD peers (2.5 versus 1.6, *P* < 0.01) and children in both groups did not show significant differences in their daily intake of grains, vegetables, and sweets [19]. A study using a case-control design found that 105 children with ASD ate more legumes (beta = 39.36, 95% CI = 24.02–54.69, *P* < 0.001) and vegetables (beta = 28.29, 95% CI = 11.61–44.97, *P* < 0.001) than TDC after adjusting for age and sex [20]. However, another case-control study showed that 53 children with ASD consumed significantly fewer daily servings of fruits and vegetables than 58 TDC (3.1 versus 4.4, *P* = 0.006) [9]. Therefore, more studies with larger sample sizes, carefully controlling for confounders, are needed to determine the differences that exist in which food groups.

To date, comparisons to control groups have identified dietary intake differences related to autism, and which autistic traits associated with the specific diets within autism are understudied. The autism-linked traits (including RRBs, sensory sensitivity, and feeding problems) are integral in our understanding of autism, not just as the specific symptoms but also in their associations with dietary and/or GI symptoms. It is reported that the more severe ASD symptoms, RRBs, and sensory sensitives, the greater food selectivity [7, 21, 22]. Specifically, repetitive patterns of behavior and interests in ASD may play a part in the development of feeding difficulties [7, 23, 24]. Sensory sensitivities in ASD may be one factor contributing to the mealtime behaviors, indicating that taste, smell, and texture play important sensory roles in accepting or rejecting food [7, 25, 26]. However, few studies have taken these traits into account and analyzed them simultaneously [17, 20, 22], and little is known about their relative contribution to dietary intake. Given the heterogeneity of autism [27] and the discrepancies in the prevalence of feeding difficulties among children with ASD [6], it is essential to determine the relative contributions of distinctive autism-linked traits to dietary intake in ASD. This will help identify the significant contributing factors to unbalanced diets and mitigate the risk of nutritional complications.

In addition to concomitant feeding problems, higher rates of co-occurring GI symptoms (e.g., constipation, diarrhea, and abdominal pain) among children with ASD have sparked significant concern [28]. Co-occurring GI complaints are serious due to the pain, discomfort, and functional impairments they produce and their short- and long-term implications for health [29]. Notably, not all studies have reported co-occurring GI conditions in ASD [30], despite the previous findings suggesting that the gut and microbiota-gut-brain axis might be involved in the pathophysiology of ASD [31]. A large autism stool metagenomics study (*n* = 247) failed to replicate previously reported associations between ASD diagnosis and microbiome, and they concluded that a less-diverse diet that relates to autism diagnostic features reduced microbial taxonomic diversity and looser stool consistency [32]. Although not direct findings of diets and GI issues, this study provided clues about the relationship between a less varied diet and GI symptoms in autism.

In the present study, we hypothesized that dietary intake and GI symptoms are altered in children with ASD when compared to age-matched TDC. In the ASD group, distinct autism-linked traits have different degrees of contribution to dietary intake, and ASD-related diets may be the main contributor to GI symptoms. The aims of this study are to: (i) compare the differences in dietary intake and GI symptoms between children with ASD and age-matched TDC. (ii) quantify the relative contributions of autism-linked traits to dietary intake, and relative contributions of autism-linked traits and dietary intake to GI symptoms in ASD children.

## Methods

### Study participants

**Fig. 1:**
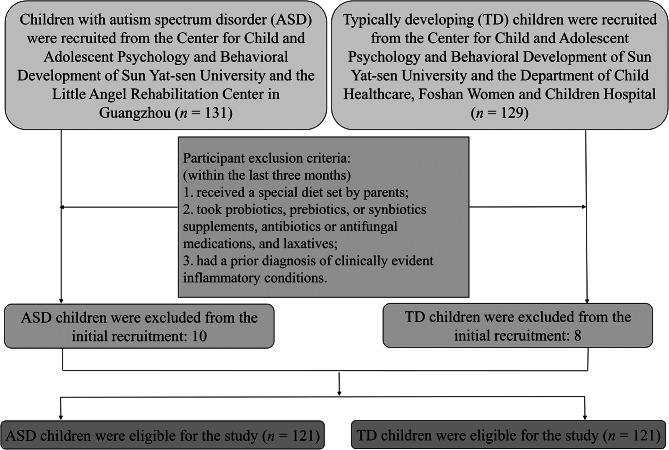
Flow diagram for study participants

An age- and sex-matched (1:1) case-control study was carried out in China. Participants were recruited based on the Research Project (Follow-up Assessment of Child Cognitive Development) between September 2021 and May 2022. Thereinto, the children with ASD were randomly recruited from the Center for Child and Adolescent Psychology and Behavioral Development of Sun Yat-sen University and Little Angel Rehabilitation Center in Guangzhou, Guangdong, China. The TD children were randomly recruited from the Center for Child and Adolescent Psychology and Behavioral Development of Sun Yat-sen University and the Department of Child Healthcare, Foshan Women and Children Hospital. The participant age range selected for this study was from 2 to 10 years to avoid breastfeeding or formula feeding as the most important source of nutrition before two years, to avoid binge eating and binge drinking, and to avoid eating outside the house during puberty (after age 10) [33]. Puberty is a critical risk period for the development of binge, loss of control eating, and more obesogenic food consumption patterns for boys and girls [34]. This study excluded children who received a special diet (including the gluten-free/casein-free diet and the ketogenic diet) set by parents, who took probiotics, prebiotics, or synbiotics supplements, antibiotics or antifungal medications, and laxatives within the last three months, and children who had a prior diagnosis of clinically evident inflammatory conditions (e.g., Crohn’s disease and ulcerative colitis). Finally, a sample of 121 children with ASD and 121 age-matched TDC were eligible for the study, see Fig. 1.

To ensure the validity and reliability of the survey data, we compared the demographic information between 121 ASD children recruited for this study and 93 ASD children recruited from July 2018 to May 2019. The results displayed that no significant differences existed in the child’s sex, ASD symptom severity, intellectual functioning, maternal educational attainment, and monthly per-capita income between these two groups of children with ASD (*P* > 0.05), implying the good representativeness of the study samples, see Supplementary Table 1 for further details.

### Sample size

Feeding problems have been found to occur in approximately 25% of TD children [35]. Children with ASD are two times more likely to experience inadequate nutrient intake than TD children [36]. The correlation coefficient between cases and their matched controls (phi) was 0.2 [37]. By taking into account the 95% qualified rate of questionnaire filling and achieving 90% statistical power (1-β) with a significance level (α) of 5%, a sample of 121 cases and 121 controls were needed in the matched case-control phase. Beyond the case-control approach, the sample size within the ASD group was calculated based on the cross-sectional study design. Specifically, the estimated prevalence of feeding problems was 80% in children with ASD [35]. In this case, we defined the allowable error as 0.1 of the prevalence estimation, and that was 8%. Therefore, 121 children with ASD were needed to obtain a 95% confidence level.

### Procedures

Parents were required to provide proof of ASD diagnosis by a licensed medical provider (e.g., psychiatrist, neurodevelopmental pediatrician, or psychologist). The patient’s ASD diagnosis was further confirmed in accordance with the DSM-5 criteria by two experienced developmental and behavioral pediatricians in this study.

Children undertook face-to-face measures by trained psychometrists or research assistants at the research center. Autism-linked traits and information on demographics, sleep, physical activity, parenting behavior, dietary intake, and GI symptoms were obtained via validated scales/questionnaires and in-person interviews with parents of children. Informed consent was obtained for participation, and the study was conducted in accordance with the principles of the Declaration of Helsinki and the Ethical Committee of the School of Public Health, Sun Yat-Sen University (No. 067 [2022]).

### Dietary assessment

Parents were also asked to complete a validated 7-day food frequency questionnaire (FFQ) [38] about their children’s consumption frequency as the number of times during the past seven days for each food, and the average quantity of food consumed each time based on a specified standardized portion size (a serving = 50 g). Then, the average daily intake of food was calculated as (consumption frequency [times/week] × average quantity each time/7). The FFQ consisted of food lists (cereals/potatoes, vegetables, fruit, livestock/poultry meat, fish/shrimp, eggs, soybeans, dairy, and drinking water).

The dietary intake indicators for this study included food groups intakes, food variety, and diet quality. The diet quality was quantified using DBI, which was established based on the recommended daily food intake from the CDGs (2022) and CFP (Supplementary Table 2). The scoring method and evaluation index of DBI were established with reference to the DBI_16 [39], see Supplementary Tables 3, 4, and 5.

### GI symptoms assessment

A shortened version of the 6-item Gastrointestinal Severity Index (6-GSI) questionnaire was intended to assess the severity of GI symptoms among patients with ASD in the present study. In a 2011 study, it was employed as an instrument to measure aspects of GI symptoms in ASD research [40]. The 6-GSI is composed of six symptoms, including constipation, diarrhea, stool consistency, stool smell, flatulence, and abdominal pain. Each symptom is rated on a three-point Likert-type scale ranging from 0 to 2, with a higher score signifying more severe GI symptoms [41]. The interrater reliability (intraclass correlation [ICC]) of the GI total scores was found to be high at 0.95 [95% CI: 0.87–0.98] [41].

### Measures of autism-linked traits

#### ASD symptom severity

The Childhood Autism Rating Scale (CARS) is a behaviorally based clinical scale derived from interaction and observation [42]. It contains 15 items rated from 1 (age appropriate) to 4 (severely autistic), covering language and communication skills, response to sensory information, and socio-emotional and interactional skills. CARS had demonstrated a high degree of internal consistency with a Cronbach’s alpha of 0.92.

and high validity with a sensitivity of 87.5% and a specificity of 90% [43]. The CARS was applied in this study to distinguish between mild-to-moderate and severe ASD. According to the manual, a CARS score between 30 and 36.5 is indicative of mild-to-moderate ASD, and a score between 37 and 60 represents severe ASD [44].

#### Restricted repetitive behaviors

The Repetitive Behavior Scale-Revised (RBS-R) is a parent or caregiver-report scale for capturing the breadth of RRBs observed in individuals with ASD. The RBS-R has sound psychometric characteristics. Cronbach’s alphas for all of the subscales were satisfactorily high (between 0.78 and 0.91, mean = 0.83) [45]. The Chinese version of the RBS-R was revised by Li et al., and it has been proved to have good reliability and validity among children with ASD [46]. The 43 items of RBS-R have been conceptually grouped into six subscales that each represents the principal typologies of restricted repetitive behaviors: stereotypic behavior, self-injurious behavior, compulsive behavior, ritualistic behavior, sameness behavior, and restricted interests. Every item is rated on a 0-point (never) to 3-point (always) Likert-type scale; thus, higher scores reflect more severe stereotypical and repetitive behavior.

### Sensory profiles

The short sensory profile (SSP), an abbreviated form of Dunn’s Sensory Profile, is one of the primary measures of sensory phenotyping among autistic children [47]. It has adequate internal consistency with Cronbach’s alpha coefficient ranging from 0.70 to 0.90 [48]. The validated Chinese version of SSP has been established by Xu et al. with good reliability and validity [49]. The test-retest reliability coefficient of the SSP is above 0.7, and the correlation coefficient between each of the subscales and the total SSP is above 0.5 [49]. The SSP consists of 38 items divided into seven subscales, each corresponding to one of the sensory subtypes: tactile, taste/smell, movement, underresponsive/seeks sensation, visual/auditory sensitivity, auditory filtering, and low energy/weak. Each item is answered on a five-point Likert-type scale ranging from always (1) to never (5), with higher total and subscale scores indicating more typical performance, while low scores emblematize heightened sensitivity in that sensory domain.

### Mealtime behaviors

The Brief Autism Mealtime Behaviour Inventory (BAMBI) is a standardized and parent-reported measure of mealtime behaviors in ASD children with adequate reliability based on internal consistency scores (Cronbach’s alpha coefficient was 0.88) [50]. A test-retest reliability coefficient was calculated between the initial administration of the BAMBI and the second administration using the total score. It was found to be significant with r of 0.87 [50]. For the criterion-related validity, there existed a significant correlation between the BAMBI total frequency score and the Behavioral Pediatric Feeding Assessment Scale (BPFAS) child behavior frequency score with r of 0.77 [50]. The BAMBI contains 18 items ranked on a five-point Likert level ranging from 1 (never/rarely) to 5 (almost every meal) and is divided into three subscales that include limited variety, food refusal, and features of ASD. The items were summed to create total scores and subscale scores. Higher scores stand for a greater frequency of problematic behaviors.

### Statistical analyses

Continuous variables were reported as mean with standard deviation (SD) or median with interquartile range (IQR), and categorical variables were expressed as numbers (percentages). Variables in the aspects of demographic information, autism-linked traits, dietary intake, and GI symptoms were compared between the ASD and TDC groups by applying the two-sample t-tests, Mann-Whitney U tests, *χ*^*2*^ tests, and multiple linear regression analysis. The relative contributions of autism-linked traits to dietary intake, and relative contributions of autism-linked traits and dietary intake to GI symptoms were estimated via the standardized beta coefficients with 95% CIs derived from the linear mixed-effects models, and the odds ratios (ORs) with 95% CIs from the mixed-effects logistic regression models. Considering the sample size, all the independent variables were not put into the mixed-effects models simultaneously but entered the mixed-effects models after carefully choosing candidate variables, to ensure parsimony of the final model. The specific screening process is as follows: (1) Candidate variables with a *P* value < 0.1 on univariate analysis and *P* value < 0.05 on multivariable models (including independent variable and covariates) (Supplementary Table 6) were put into the multivariable models simultaneously (Supplementary Table 7) to screen out final variables. (2) Final variables showing significant associations (*P* value < 0.05) with different dietary and GI outcomes were put into the multivariable mixed-effects models simultaneously to compare the relative contributions of autism-linked traits. Notably, collinearity diagnosis was conducted to test the potential multicollinearity among the variables (independent and covariates variables) included in the models. To estimate effect sizes, the formula r = Z/√(N) was used for Mann-Whitney U-tests, effect size φ for 2 × 2 tables, and Cramer-V for tables larger than 2 × 2, with threshold values of 0.1, 0.3, and 0.5 used to categorize effects as small, medium, and large, respectively [51]. The Cohen’s f^2^ was calculated for multiple regression models, and f^2^ ≥ 0.02, f^2^ ≥ 0.15, and f^2^ ≥ 0.35 were interpreted as small, medium, and large effect sizes, respectively [52].

Also, one-to-one propensity score matching (PSM) was performed to create a balanced covariate distribution between ASD and TD groups based on the nearest neighbor method, and then the dietary intake and GI symptoms were compared between the ASD and TD sub-groups. The variables selected for the propensity score calculation included the child’s age, sex, intellectual functioning, birth mode, birth order, average daily sleep duration, maternal educational attainment, gestational diabetes mellitus, maternal obesity, and monthly per-capita income [52–57], and the score was calculated using a logistic regression model. Details of covariates are presented in Supplementary Table 8.

All statistical analyses were carried out using SPSS Statistics 26.0 (IBM Corp. Released 2019. IBM SPSS Statistics for Windows, Version 26.0. Armonk, NY, USA: IBM Corp) and GraphPad Prism version 8.4.2 (GraphPad Software, San Diego, CA, USA). Statistical significance was set at a two-tailed *P* < 0.05.

## Results

There were statistically significant differences between ASD and TDC groups in the demographic characteristics, including sex, intellectual functioning, birth mode, birth order, average daily sleep duration, maternal educational attainment, gestational diabetes mellitus, maternal obesity, and monthly per-capita income (*P* < 0.05, effect size > 0.1), see Table 1.

**Table 1:** Demographic characteristics of the children with ASD and age-matched TD children

Variables	ASD (*n* = 121)	TD (*n* = 121)	P value	Effect size
Child characteristics				
Age, median (IQR), y	6.3 (4.8, 7.3)	6.8 (3.6, 7.9)	0.810	0.016
Sex, n (%)				
Male	98 (81)	82 (67.8)	0.018	0.152
Female	23 (19)	39 (32.2)		
Height, median (IQR), cm	118.6 (105, 127.9)	119.2 (100.1, 130)	0.864	0.011
Weight, median (IQR), kg	21.2 (16, 26.8)	21.2 (15, 26.6)	0.417	0.052
BMI, median (IQR), kg/m^2^	15.4 (14.4, 16.7)	15.3 (14.4, 16.3)	0.382	0.056
ASD symptom severity^a^, n (%)				
Mild-to-moderate	98 (81)	NA	NA	NA
Severe	23 (19)	NA	NA	NA
Intellectual functioning^b^, n (%)				
Normal	55 (45.5)	104 (86)	< 0.001	0.334
Borderline	14 (11.6)	12 (9.9)		
Abnormal	52 (43)	5 (4.1)		
Premature birth, n (%)				
Yes	8 (6.6)	6 (5)	0.582	0.035
No	113 (93.4)	115 (95)		
Birth mode, n (%)				
Vaginal delivery	72 (59.5)	74 (61.2)	0.002	0.164
Elective caeserean section	21 (17.4)	37 (30.6)		
Emergency caeserean section	28 (23.1)	10 (8.3)		
Feeding patterns before 2 years of age^c^, n (%)				
Breastfeeding	28 (23.1)	27 (22.3)	0.956	0.014
Mixed feeding	30 (24.8)	32 (26.4)		
Artificial feeding	63 (52.1)	62 (51.2)		
Birth order, n (%)				
1	73 (60.3)	91 (75.2)	0.013	0.159
> 1	48 (39.7)	30 (24.8)		
Sleep duration, median (IQR), hours/day	9.7 (9.1, 10.6)	10.3 (9.4, 11.1)	0.001	0.207
SB, median (IQR), hours/d	2.7 (1.3, 4.1)	3.6 (1.0, 5.6)	0.065	0.118
MVPA, median (IQR), hours/d	0.7 (0.3, 1.1)	0.8 (0.4, 1.4)	0.240	0.076
Walking, median (IQR), hours/d	0.5 (0.3, 1.0)	0.5 (0.2, 0.9)	0.476	0.046
**Maternal Characteristics**				
Advanced maternal age, n (%)				
Yes	7 (5.8)	10 (8.3)	0.450	0.049
No	114 (94.2)	111 (91.7)		
Educational attainment, n (%)				
College degree or below	67 (55.4)	37 (30.6)	< 0.001	0.250
Undergraduate degree or higher	54 (44.6)	84 (69.4)		
Gestational hypertension, n (%)				
Yes	2 (1.7)	2 (1.7)	1.000	0.000
No	119 (98.3)	119 (98.3)		
Gestational diabetes mellitus, n (%)				
Yes	21 (17.4)	10 (8.3)	0.034	0.136
No	100 (82.6)	111 (91.7)		
Maternal obesity, n (%)				
Yes	9 (7.4)	2 (1.7)	0.031	0.139
No	112 (92.6)	119 (98.3)		
**Family characteristics**				
Monthly per-capita income, n (%)				
≤ 8,000 RMB	86 (71.1)	54 (44.6)	0.011	0.163
> 8,000 RMB	35 (28.9)	67 (55.4)		
Parenting behavior^d^, n (%)				
Support/engagement	110 (90.9)	113 (93.4)	0.473	0.046
Opposition/defiance	11 (9.1)	8 (6.6)		

^a^ Evaluated by the Childhood Autism Rating Scale (CARS). ^b^ Evaluated by the Wechsler Intelligence Scale for Children Forth Edition (WISC-IV) or Gesell developmental Schedules (GDS). ^c^ Longest sustained feeding patterns before 2 years of age. ^d^ Evaluated by the Parent Behavior Inventory (PBI). Effect size = Z/√(N) for Mann–Whitney U-tests, and effect size φ or Cramer-V for *χ*^*2*^ tests. ASD: autism spectrum disorder, TD: typically developing, IQR: interquartile range, BMI: body mass index, SB: sedentary behavior, MVPA: moderate-to-vigorous physical activity, RMB: Ren Min Bi (Chinese currency), NA: not applicable

### Children with ASD had poorer diets than age-matched TD children

**Fig. 2:**
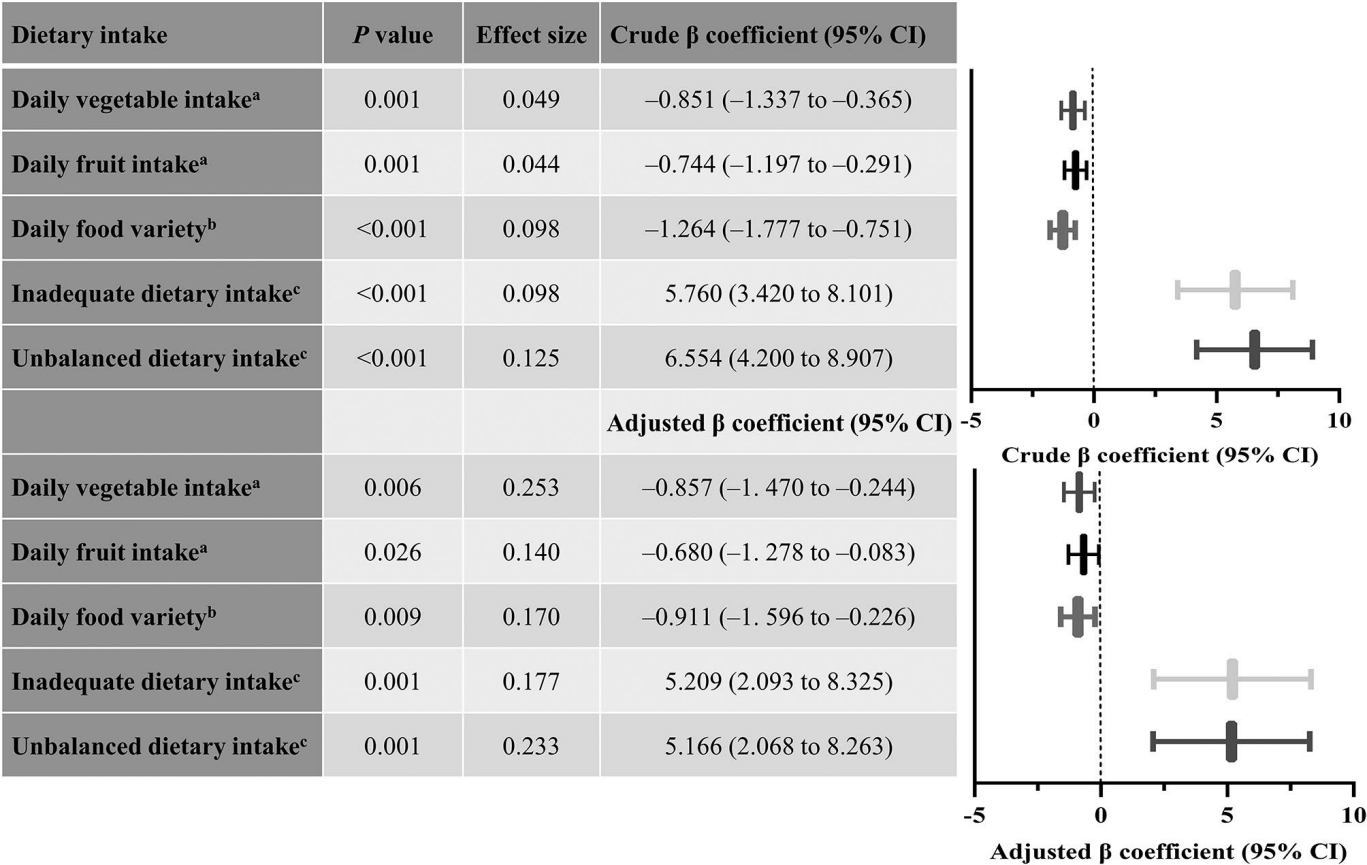
Children with ASD had poorer diets than age-matched TD children (*n* = 242).^a^ g/day. ^b^ Food variety scores ranged from − 12 to 0, with lower scores representing less variety of food. ^c^ Evaluated using the low bound score (LBS) and diet quality distance (DQD), respectively, with higher scores representing a higher degree of inadequate/unbalanced diets. Adjusted for child’s age, sex, intellectual functioning, birth mode, birth order, average daily sleep duration, maternal educational attainment, gestational diabetes mellitus, maternal obesity, monthly per-capita income, and parenting behavior. Effect size = Cohen’s f^2^ for linear models. ASD: autism spectrum disorder, TD: typically developing, CI: confidence interval

There were significant differences between ASD and TDC groups in the daily vegetables/fruit intake, daily food variety, and inadequate/unbalanced dietary intake (*P* < 0.05, effect size > 0.1), and the results remained consistent when the participants were stratified by age into 2–5 and 6–10 years old, see Supplementary Table 9. When the participants were matched using PSM, children with ASD had poorer diets with lower daily vegetables/fruit intake, less variety of food, and a higher degree of inadequate/unbalanced dietary intake (*P* < 0.05, effect size > 0.1), see Supplementary Table 11. Similarly, the crude and adjusted general linear models show that children with ASD had poorer diets than age-matched TDC (*P* < 0.05, effect size > 0.02), see Fig. 2.

### Children with ASD experienced more severe GI symptoms than age-matched TD children

**Fig. 3:**
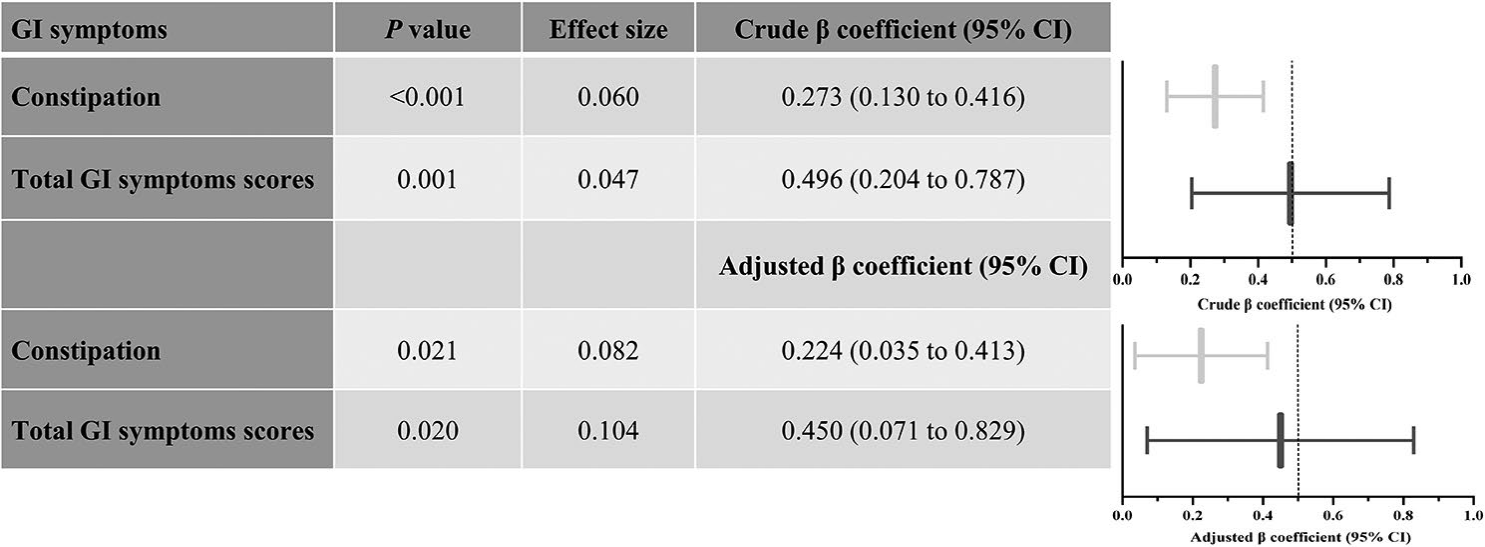
Children with ASD experienced more severe GI symptoms than age-matched TD children (*n* = 242). Constipation and total GI symptoms scores were evaluated by subscale and total scores of the 6-item gastrointestinal severity index (6-GSI). Adjusted for child’s age, sex, intellectual functioning, birth mode, birth order, average daily sleep duration, maternal educational attainment, gestational diabetes mellitus, maternal obesity, and monthly per-capita income. Effect size = Cohen’s f^2^ for linear models. GI: gastrointestinal, ASD: autism spectrum disorder, TD: typically developing, CI: confidence interval

There were significant differences between the ASD and TDC groups in constipation, abnormal smell of stool, and total GI symptoms scores (*P* < 0.05, effect size > 0.1), see Supplementary Table 10. When the participants were matched using PSM, the children with ASD suffered far more severe constipation and total GI symptoms (*P* < 0.05, effect size > 0.1), see Supplementary Table 11. Similarly, the crude and adjusted general linear models show that children with ASD experienced more severe constipation and total GI symptoms than age-matched TDC (*P* < 0.05, effect size > 0.02), see Fig. 3.

### The relative contribution of autism-linked traits to poorer diets in children with ASD

The significant differences between ASD and TDC groups in sensory profiles and mealtime behaviors were identified before assessing the relative contributions of autism-linked traits to diets (*P* < 0.05, effect size > 0.1), see Supplementary Table 12. Similarly, the crude and adjusted general linear models show that children with ASD experienced more severe sensory abnormalities and mealtime behavior problems (including subtype limited variety) than age-matched TDC (*P* < 0.05, effect size > 0.02), see Supplementary Tables 13 and 14.

**Fig. 4:**
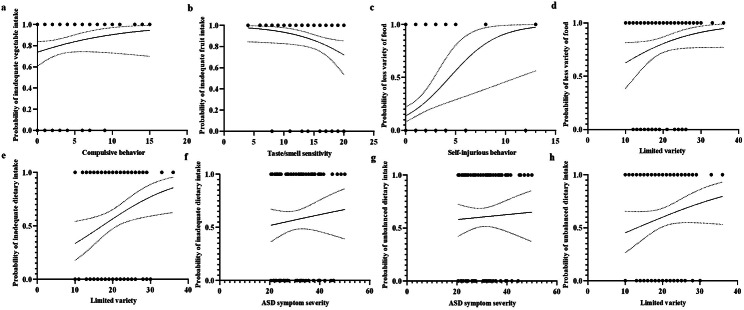
Probability of poorer diets associated with autism-linked traits in children with ASD (*n* = 121). **a**, **b** Inadequate vegetable/fruit intake: inadequate vs. appropriate intake; **c**, **d** Less variety of food: food variety scores between − 12 to − 7 vs. − 6 to 0 (with lower scores representing less variety of food); **e**, **f** Inadequate dietary intake: moderate-high inadequate dietary intake vs. appropriate, more appropriate, and low inadequate dietary intake; **g**, **h** Unbalanced dietary intake: moderate-high unbalanced dietary intake vs. more appropriate, and low unbalanced dietary intake. ASD: autism spectrum disorder

**Fig. 5:**
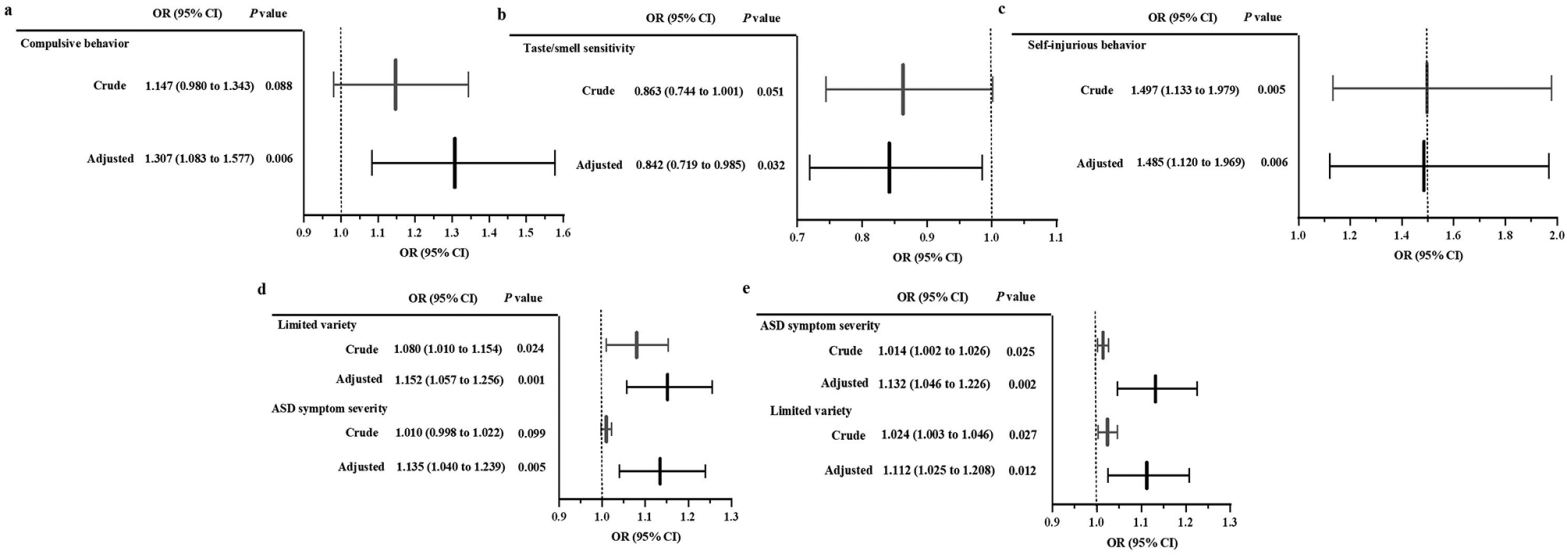
The relative contribution of autism-linked traits to the odds of poorer diets in children with ASD (*n* = 121). **a**, **b** Inadequate vegetable/fruit intake: inadequate vs. appropriate intake; **c** Less variety of food: food variety scores between − 12 to − 7 vs. − 6 to 0 (with lower scores representing less variety of food); **d** Inadequate dietary intake: moderate-high inadequate dietary intake vs. appropriate, more appropriate, and low inadequate dietary intake; **e** Unbalanced dietary intake: moderate-high unbalanced dietary intake vs. more appropriate, and low unbalanced dietary intake. Adjusted for child’s age, sex, intellectual functioning, birth mode, average daily sleep duration, average daily SB time, average daily MVPA time, average daily walking time, maternal educational attainment, monthly per-capita income, and parenting behavior. ASD: autism spectrum disorder, OR: odds ratio, CI: confidence interval, SB: sedentary behavior, MVPA: moderate-to-vigorous physical activity

The crude and adjusted associations of autism-linked traits with dietary intake in the ASD group are displayed in Table 2, Fig. 4, and Fig. 5. Figure 4 is the probability plot in mixed-effects logistic regression models for assessing the probability of poorer diets associated with autism-linked traits. The linear mixed-effects models (Table 2) and mixed-effects logistic regression models (Fig. 5) show the relative contributions of autism-linked traits to poorer diets. Only compulsive behavior (a subtype of RRBs) was statistically significantly associated with lower daily vegetable intake (adjusted standardized β: − 0.291, 95% CI: − 0.504 to − 0.077, *P* = 0.008, effect size = 0.267), and was statistically significantly associated with higher odds of inadequate vegetable intake (adjusted OR: 1.307, 95% CI: 1.083 to 1.577, *P* = 0.006). Only taste/smell sensitivity (a subtype of sensory symptoms) was significantly associated with lower daily fruit intake (adjusted standardized β: 0.240, 95% CI: 0.009 to 0.470, *P* = 0.042, effect size = 0.175), and was significantly associated with higher odds of inadequate fruit intake (adjusted OR: 0.842, 95% CI: 0.719 to 0.985, *P* = 0.032). Only self-injurious behavior (a subtype of RRBs) was significantly associated with less variety of food (adjusted standardized β: − 0.228, 95% CI: − 0.432 to − 0.024, *P* = 0.029, effect size = 0.280), and was statistically significantly associated with higher odds of less food variety (adjusted OR: 1.485, 95% CI: 1.120 to 1.969, *P* = 0.006). Limited variety (adjusted standardized β: 0.289, 95% CI: 0.063 to 0.514, *P* = 0.013, effect size = 0.304) was a bigger contributor than ASD symptom severity (adjusted standardized β: 0.261, 95% CI: 0.005 to 0.517, *P* = 0.046, effect size = 0.304) to inadequate dietary intake. Also, limited variety (adjusted OR: 1.152, 95% CI: 1.057 to 1.256, *P* = 0.001) played a bigger role in higher odds of inadequate dietary intake relative to ASD symptom severity (adjusted OR: 1.135, 95% CI: 1.040 to 1.239, *P* = 0.005). ASD symptom severity (adjusted standardized β: 0.347, 95% CI: 0.092 to 0.601, *P* = 0.008, effect size = 0.350) was a bigger contributor than limited variety (adjusted standardized β: 0.312, 95% CI: 0.093 to 0.531, *P* = 0.006, effect size = 0.350) to unbalanced dietary intake; ASD symptom severity (adjusted OR: 1.132, 95% CI: 1.046 to 1.226, *P* = 0.002) played a bigger role in higher odds of unbalanced dietary intake relative to limited variety (adjusted OR: 1.112, 95% CI: 1.025 to 1.208, *P* = 0.012).

**The relative contribution of autism-linked traits and unbalanced dietary intake to GI symptoms in children with ASD**.

The crude and adjusted associations of autism-linked traits and dietary intake with GI symptoms in children with ASD are analyzed. The linear mixed-effects models (Table 3) show that unbalanced dietary intake was a significant independent factor associated with constipation (adjusted standardized β: 0.198, 95% CI: 0.023 to 0.374, *P* = 0.027, effect size = 0.214) and total GI symptoms scores (adjusted standardized β: 0.231, 95% CI: 0.063 to 0.400, *P* = 0.008, effect size = 0.284), and no statistically significant associations between autism-linked traits and constipation/total GI symptoms scores were identified in children with ASD.

**Table 2:** The relative contribution of autism-linked traits to poorer diets in children with ASD (*n* = 121)

Autism-linked traits	Daily vegetable intake^a^				Daily fruit intake^a^						Daily food variety^b^				
	Crude β coefficient (95% CI)	*P* value	Effect size		Crude β coefficient (95% CI)			*P* value	Effect size		Crude β coefficient (95% CI)			*P* value	Effect size
ASD symptom severity	0.050 (–0.131 to 0.232)	0.584	0.003		–0.057 (–0.239 to 0.124)			0.533	0.003		–0.128 (–0.308 to 0.053)			0.163	0.016
Self-injurious behavior	–0.115 (–0.295 to 0.065)	0.209	0.013		–0.140 (–0.320 to 0.040)			0.125	0.020		–0.301 (–0.475 to − 0.128)			0.001	0.100
Compulsive behavior	–0.245 (–0.421 to − 0.069)	0.007	0.064		–0.052 (–0.233 to 0.130)			0.572	0.003		–0.177 (–0.355 to 0.002)			0.053	0.032
Taste/smell sensitivity	0.178 (–0.001 to 0.357)	0.050	0.033		0.247 (0.071 to 0.423)			0.006	0.065		0.289 (0.115 to 0.462			0.001	0.091
Limited variety	–0.250 (–0.426 to − 0.074)	0.006	0.066		–0.141 (–0.321 to 0.038)			0.122	0.020		–0.279 (–0.453 to − 0.104)			0.002	0.085
	**Inadequate dietary intake** ^**c**^							**Unbalanced dietary intake** ^**c**^							
	**Crude β coefficient (95% CI)**	***P*** **value**			**Effect size**			**Crude β coefficient (95% CI)**				***P*** **value**		**Effect size**	
ASD symptom severity	0.028 (–0.154 to 0.209)	0.764			0.001			0.055 (–0.126 to 0.236)				0.548		0.003	
Self-injurious behavior	0.168 (–0.011 to 0.347)	0.066			0.029			0.167 (–0.013 to 0.346)				0.068		0.029	
Compulsive behavior	0.075 (–0.106 to 0.256)	0.416			0.006			0.053 (–0.128 to 0.234)				0.564		0.003	
Taste/smell sensitivity	–0.238 (–0.415 to − 0.062)	0.008			0.061			–0.231 (–0.408 to − 0.054)				0.011		0.056	
Limited variety	0.229 (0.053 to 0.406)	0.011			0.056			0.233 (0.056 to 0.410)				0.010		0.057	
	**Daily vegetable intake** ^**a**^				**Daily fruit intake** ^**a**^						**Daily food variety** ^**b**^				
	**Adjusted β coefficient (95% CI)**	***P*** **value**	**Effect size**		**Adjusted β coefficient (95% CI)**			***P*** **value**	**Effect size**		**Adjusted β coefficient (95% CI)**			***P*** **value**	**Effect size**
ASD symptom severity	0.201 (–0.044 to 0.445)	0.097	0.267		–0.053 (–0.276 to 0.171)			0.631	0.175		–0.091 (–0.335 to 0.154)			0.450	0.280
Self-injurious behavior	–0.048 (–0.253 to 0.156)	0.641			–0.090 (–0.302 to 0.122)			0.402			–0.228 (–0.432 to − 0.024)			0.029	
Compulsive behavior	–0.291 (–0.504 to − 0.077)	0.008			0.057 (–0.158 to 0.271)			0.600			–0.013 (–0.221 to 0.194)			0.899	
Taste/smell sensitivity	–0.030 (–0.254 to 0.194)	0.792			0.240 (0.009 to 0.470)			0.042			0.091 (–0.133 to 0.314)			0.423	
Limited variety	–0.206 (–0.426 to 0.013)	0.065			0.012 (–0.213 to 0.237)			0.917			–0.195 (–0.416 to 0.025)			0.082	
	**Inadequate dietary intake** ^**c**^							**Unbalanced dietary intake** ^**c**^							
	**Adjusted β coefficient (95% CI)**	***P*** **value**			**Effect size**			**Adjusted β coefficient (95% CI)**					***P*** **value**	**Effect size**	
ASD symptom severity	0.261 (0.005 to 0.517)	0.046			0.304			0.347 (0.092 to 0.601)					0.008	0.350	
Self-injurious behavior	0.151 (–0.052 to 0.355)	0.144						0.139 (–0.060 to 0.338)					0.169		
Compulsive behavior	0.004 (–0.201 to 0.209)	0.972						–0.032 (–0.235 to 0.171)					0.756		
Taste/smell sensitivity	–0.017 (–0.242 to 0.209)	0.883						0.002 (–0.218 to 0.222)					0.983		
Limited variety	0.289 (0.063 to 0.514)	0.013						0.312 (0.093 to 0.531)					0.006		

^a^ g/day. ^b^ Food variety scores ranged from − 12 to 0, with lower scores representing less variety of food. ^c^ Evaluated using the low bound score (LBS)/diet quality distance (DQD), with higher scores representing a higher degree of inadequate/unbalanced diets. ASD symptom severity was evaluated by the Childhood Autism Rating Scale (CARS). Restricted repetitive behaviors, sensory profiles, and mealtime behaviors were evaluated by subscale and total scores of repetitive behavior scale-revised (RBS-R), short sensory profile (SSP), and brief autism mealtime behavior inventory (BAMBI), respectively. Adjusted for child’s age, sex, intellectual functioning, birth mode, average daily sleep duration, average daily SB time, average daily MVPA time, average daily walking time, maternal educational attainment, monthly per-capita income, and parenting behavior. Effect size = Cohen’s f^2^ for mixed-effects models. ASD: autism spectrum disorder, CI: confidence interval, SB: sedentary behavior, MVPA: moderate-to-vigorous physical activity

**Table 3:** The relative contribution of autism-linked traits and unbalanced dietary intake to GI symptoms in children with ASD (*n* = 121)

Dietary intake	Constipation				Total GI symptoms scores		
	Crude β coefficient (95% CI)	*P* value	Effect size		Crude β coefficient (95% CI)	*P* value	Effect size
Unbalanced dietary intake	0.187 (0.008 to 0.365)	0.040	0.036		0.202 (0.024 to 0.379)	0.027	0.043
	**Constipation**				**Total GI symptoms scores**		
	**Adjusted β coefficient (95% CI)**	***P*** **value**	**Effect size**		**Adjusted β coefficient (95% CI)**	***P*** **value**	**Effect size**
Unbalanced dietary intake	0.198 (0.023 to 0.374)	0.027	0.214		0.231 (0.063 to 0.400)	0.008	0.284

Unbalanced dietary intake was evaluated by the diet quality distance (DQD). Constipation and total GI symptoms scores were evaluated by subscale and total scores of the 6-item gastrointestinal severity index (6-GSI). Adjusted for child’s age, sex, intellectual functioning, birth mode, average daily sleep duration, average daily SB time, average daily MVPA time, average daily walking time, maternal educational attainment, monthly per-capita income, and parenting behavior. Effect size = Cohen’s f^2^ for mixed-effects models. GI: gastrointestinal, ASD: autism spectrum disorder, CI: confidence interval, SB: sedentary behavior, MVPA: moderate-to-vigorous physical activity

## Discussion

In this study, children with ASD ate poorer diets with lower vegetables/fruit, less variety of food, a higher degree of inadequate/unbalanced dietary intake, and experienced more severe constipation/total GI symptoms than age-matched TDC. Among the ASD group, ASD symptom severity, compulsive and self-injurious behavior, taste/smell sensitivity, and limited variety were the top contributors to poorer diets. Notably, unbalanced dietary intake was a significant independent factor associated with constipation/total GI symptoms in ASD, and autism-linked traits showed no contributions.

Making comparisons of dietary differences between ASD and age-matched TD groups after adjusting for a comprehensive set of covariates was a highlight of this study. Regarding the specific food groups, our findings were consistent with most of the previous studies that autistic children consumed fewer vegetables/fruits than TD children [14, 58–62]. However, contrasting findings of higher vegetables/fruit consumption in the ASD group or equal amounts of vegetables/fruit consumption in both groups were revealed in some previous research [20, 63–65]. This is probably because of the food cultural and national diversity among participants and the different definitions of vegetables/fruit, for example, some vegetables included both raw and boiled and some fruits included fresh fruit juice [59, 65, 66]. In our study, apart from lower vegetables/fruit consumption, a less variety of food and a higher degree of inadequate/unbalanced diets among ASD children were observed. In line with our findings, a study [65] from Hong Kong with similar traditional Chinese culture recorded that there was less variety of food and poorer overall diet quality in children with ASD than in the TD group. Prior evidence supported these findings, indicating that children with ASD had a more limited food repertoire compared to TD children (19.0 foods vs. 22.5 foods)., as well as inadequate intake of a greater number of nutrients (with or without the exclusion of children on special diets) [67]. Similarly, Sharp et al. found that 78.5% of children with ASD followed a diet at risk for five or more nutrient inadequacies [14]. On the contrary, a study [20] performed in the Mediterranean Region reported that the food variety score and healthy eating index were not significant between ASD and TD children (6–9 years), which may be explained by the fact that parents of children who had been diagnosed with autism pay specific attention to diets of their children and these compensate to some degree for unhealthier diets. Combined with previous studies, we discovered the poorer diet quality in children with ASD compared to TD children. The prolonged exposure to unhealthier diets deserves attention because of the adverse outcomes like developmental and growth delays, inappropriate weight gain, cognitive and social deficits, and poor academic achievement [10, 22, 68, 69].

Results of the present study indicated that compulsive behavior, a subtype of RRBs, was the only contributor to less vegetable intake in children with ASD. Genetic contributions [32] to compulsive behavior and the serotonin system [70] could be the potential explanations for this finding. The other autism-linked traits like ASD symptom severity, sensory abnormalities, and mealtime behavior problems showed no associations with vegetable consumption in the current study. Related to our findings, Marler et al. recorded that there existed an association between compulsive behavior and functional constipation in autism [71]. As we know, diets rich in vegetables can prevent and alleviate constipation because of their dietary fiber content which can provide a natural laxative effect [72]. Future research elucidating the mechanisms involved in lower vegetables intake in ASD can help us clarify these complex associations and implement the targeted interventions.

The present study also indicated that taste/smell sensitivity, a subtype of sensory abnormalities, was the only contributor to lower fruit consumption. Higher taste/smell sensitivity may limit autistic children’s willingness to experiment with fruit with a stronger flavor and odor and restrict their feeding to preferred and tolerable food. This is a novel finding, most previous studies investigated the associations between sensory sensitivity and eating behaviors rather than dietary intake [73, 74]. Despite Coulthard et al. suggesting that children with sensitivities to taste and smell ate fewer fruits, this study was conducted among TD children instead of among children with ASD [75]. Only Chistol et al. in 2017 observed similar results to ours that autistic children with atypical Oral Sensory Over-sensitivity (a measure of sensory hyper-sensitivity for taste and smell sensory input) consumed less variety of fruits than those with ASD without Oral Sensory Over-sensitivity [76]. Insufficient consumption of fruit in childhood can increase the risk of micronutrient deficiencies and a range of future chronic diseases such as cardiovascular disease and cancer [77]. Early identification of and intervention for taste/smell abnormalities is crucial for promoting greater consumption of fruit in children with ASD.

Previous evidence had proposed that the consumption of less variety of food would stem from more mealtime behaviors in children with ASD [67]. As a strength, we simultaneously analyzed the autism-linked traits to quantify their relative contributions to less variety of food. It is surprising that self-injurious behavior, a subtype of RRBs, was the only contributing trait. Similarly, self-injurious behavior has been recognized as a correlate of anorexia nervosa, and there seem to be psychopathological links between them in emotion dysregulation, maladaptive coping, and a dysfunctional reward system [78]. In this regard, a worthwhile avenue for future study is exploring the psychopathological and neurobiological overlap between self-injurious behavior and fewer kinds of food in ASD for clarifying their basic mechanisms.

With regard to inadequate dietary intake, this study provided evidence that limited variety – a subtype of mealtime behavior problems – was the primary contributing trait, followed by ASD symptom severity. Indeed, there have been suggestions that the deteriorated feeding and mealtime behaviors observed in children with ASD may correspond to a lower diet quality [79]. Interestingly, the ASD symptom severity was found to be the larger contributor than limited variety to unbalanced dietary intake in the present study. Coincidentally, Patton et al. [22] stated that greater ASD severity was associated with fewer bites of unfamiliar food, greater disruptive behavior during meals, and greater parental commands to take bites during meals; higher levels of limited variety were only associated with fewer bites of unfamiliar food. Based on this finding, it may be inferred that ASD symptom severity as a composite indicator played a more prominent role than limited variety in the children’s unbalanced dietary intake through the influence of various aspects. Priority should be given to implementing dietary assessments for children with more severe autism and limited food variety to address the health and nutritional challenges.

Consistent with previous systematic reviews and meta-analyses, our results indicated that the children with ASD suffered more severe constipation/total GI symptoms [31, 80]. ASD-related behaviors such as stereotypic or repetitive behaviors, self-injurious and aggressive behaviors, sensory over-responsivity, sleep disturbances, sudden irritability, and anxiety are very common in autistic children with GI symptoms [81–83], and thus some have suggested that these certain problematic behaviors may be the possible expressions of GI symptoms [29, 81]. However, these common problematic behaviors are not specific to those with GI symptoms but also to autistic children without GI symptoms [81]. Based on these results, we speculated that the core autistic symptoms may not be the main influential factors of GI conditions. In the current study, only unbalanced dietary intake instead of autism-linked traits was found to be associated with constipation/total GI symptoms in children with ASD. This is supported by Berding et al. [84], who raised that an eating pattern characterized by a higher intake of healthy foods such as fruit, vegetables, legumes, nuts, and seeds was associated with a bacterial profile that could potentially be linked to some aspects of GI health [84–86]. Valenzuela et al. in 2022 reported an unbalanced diet (low numbers of fruits and vegetables or foods rich in fiber) seems to have an essential role in the appearance of GI symptoms due to diet’s predominant role in changes in the gut microbiota [87]. Yap et al. in 2021 claimed that microbiome differences in ASD may reflect ASD-specific dietary preferences, whereas direct associations between the gut microbiome and ASD diagnosis were negligible [32]. In recent years, more attention has been paid to the relationship between eating behaviors and GI symptoms [88]. Leader et al. noted that more GI symptoms were reported in children and adolescents with ASD who experienced feeding problems than those who did not [89]. However, mealtime behaviors showed no significant associations with GI symptoms in our study. Also, the research conducted by Postorino found no evidence of a relationship between GI symptoms and food selectivity in children with ASD [90]. This inconsistency suggested that the problematic mealtime behaviors could not be used to explain GI problems alone and that the dietary imbalance associated with autistic traits was the key contributing factor to GI questions. On this basis, we further speculate that an unbalanced diet in children with autism may contribute to GI symptoms via changes in the microbiome and intestinal permeability [87], and we will continue to explore this direction.

The present study has several strengths. This study jointly used the linear mixed-effects model and mixed-effects logistic regression model, allowing us to examine the relative contributions of autism-linked traits to dietary and GI problems in children with ASD. The study encompassed greater numbers of participants, as well as compared dietary intake and GI symptoms based on PSM. It also controlled for a wider spectrum of covariates than most of the previous studies in ASD [32, 91], thus mitigating confounding bias. The study firstly evaluated diet quality by applying the DBI based on the CDGs (2022) and CFP. Some other strengths of this study were that all subjects were recruited based on research projects, which may help reduce the admission rate bias [92]. The present study was integrated into a comprehensive assessment project, which may help mitigate parents’ recruitment bias stemming from their interest in dietary intake and adverse GI reactions [93]. To control for the investigation bias, all investigators underwent rigorous training, two assessments, and three on-site supervision and feedback sessions, and the evaluation data and reports were reviewed by the different investigators [94]. This study also has several limitations. The questionnaires such as FFQ, International Physical Activity Questionnaire Short-Form (IPAQ-SF), and Children’s Sleep Habits Questionnaire (CSHQ) completed by parents may be subject to social desirability bias and recall bias [15, 95]. Future studies are encouraged to apply specific biochemical parameters as a surrogate for measuring dietary intake. Additionally, the use of Actigraph GT3X + accelerometer and sleep trackers can provide objective monitoring of physical activity and sleep duration. Besides, the absence of strict limits on the severity of autism symptoms may result in unequal distributions of ASD symptom severity, with a smaller sample size of severe severity. The higher prevalence of ASD in boys [16] leads to unequal gender distributions with smaller sample sizes of ASD girls observed in this study. Finally, the mechanisms underlying dietary and GI issues have not been thoroughly elucidated. Future studies should integrate microbiome and metabolome analyses to investigate these potential mechanisms in ASD. In addition to the GI symptoms, diseases such as celiac disease, inflammatory bowel disease (IBD), and functional GI disorders frequently co-exist with ASD [96–97], and future studies can also utilize a multi-omics approach to probe the comorbid mechanism of these disorders and autism.

In conclusion, children with ASD had poorer diets, more severe GI symptoms, sensory abnormalities, and mealtime behavior problems than age-matched TDC. ASD symptom severity, compulsive and self-injurious behavior, taste/smell sensitivity, and limited variety were the top contributing traits to poorer diets. An unbalanced dietary intake was a significant independent factor associated with constipation/total GI symptoms. These findings identified the different levels of contributors to dietary and GI questions which provide the largest benefits for the dietary and GI problems of ASD if they were targeted for early detection and optimal treatment.

### Electronic supplementary material

Below is the link to the electronic supplementary material.

Supplementary Material 1

Supplementary Material 2

Supplementary Material 3

## Data Availability

The datasets analyzed during the current study are available from the corresponding author upon reasonable request.

## Abbreviations

GI: gastrointestinal
ASD: autism spectrum disorder
TDC: typically developing children
RRBs: restricted repetitive behaviors
DSM-5: Diagnostic and Statistical Manual of Mental Disorders,Fifth Edition
APA: American Psychiatric Association
ADHD: attention deficit hyperactivity disorder
AD: anxiety disorders
OCD: obsessive-compulsive disorder
ADI-R: Autism Diagnostic Interview-Revised
ADOS-CSS: Autism Diagnostic Observation Scale-Calibrated Severity Scale
CDGs: Chinese Dietary Guidelines
CFP: Chinese Food Pagoda
FFQ: food frequency questionnaire
DBI: Dietary balance index
LBS: low bound score
DQD: diet quality distance
6-GSI: 6-item Gastrointestinal Severity Index
ICC: intraclass correlation
CARS: Childhood Autism Rating Scale
RBS-R: Repetitive Behavior Scale-Revised
SSP: Short Sensory Profile
BAMBI: Brief Autism Mealtime Behaviour Inventory
BPFAS: Behavioral Pediatric Feeding Assessment Scale
SD: standard deviation
IQR: interquartile range
CIs: confidence intervals
ORs: odds ratios
PSM: propensity score matching
IPAQ-SF: International Physical Activity Questionnaire Short-Form
CSHQ: Children’s Sleep Habits Questionnaires
IBD: inflammatory bowel disease
