# Correction: Dietary intake and gastrointestinal symptoms are altered in children with Autism Spectrum Disorder: the relative contribution of autism-linked traits

**DOI:** 10.1186/s12937-024-00942-4

**Published:** 2024-04-02

**Authors:** Hailin Li, Saijun Huang, Jin Jing, Hong Yu, Tingfeng Gu, Xiaoxuan Ou, Shuolin Pan, Yanna Zhu, Xi Su

**Affiliations:** 1 Department of Child Healthcare, Foshan Women and Children Hospital, Foshan, Guangdong 528000 P.R. China; 2 Research Center of Children and Adolescent Psychological and Behavioral Development, Department of Maternal and Child Health, School of Public Health, Sun Yat-sen University, Guangzhou, Guangdong 510080 P.R. China; 3 Department of Maternal and Child Health, School of Public Health, Guangdong Provincial Key Laboratory of Food, Nutrition and Health, Sun Yat-sen University, Guangzhou, Guangdong 510080 P.R. China

**Correction: Nutr J 23, 27 (2024)**

10.1186/s12937-024-00930-8

Following publication of the original article [1], the author reported that affiliations 1 and 2 should be switched.

The previous affiliations 1 and 2 are:

^1^Research Center of Children and Adolescent Psychological and Behavioral Development, Department of Maternal and Child Health, School of Public Health, Sun Yat-sen University, Guangzhou, Guangdong 510,080, P.R. China.

^2^Department of Child Healthcare, Foshan Women and Children Hospital, Foshan, Guangdong 528,000, P.R. China.

The present affiliations 1 and 2 are now:

^1^Department of Child Healthcare, Foshan Women and Children Hospital, Foshan, Guangdong 528,000, P.R. China.

^2^Research Center of Children and Adolescent Psychological and Behavioral Development, Department of Maternal and Child Health, School of Public Health, Sun Yat-sen University, Guangzhou, Guangdong 510,080, P.R. China.

The original article has been updated.
