# Supplementary material for: Dietary intake and gastrointestinal symptoms are altered in children with Autism Spectrum Disorder: the relative contribution of autism-linked traits
Source: Nutr J. 2024 Feb 28;23:27. doi: 10.1186/s12937-024-00930-8 (PMC10900601; doi:10.1186/s12937-024-00930-8)
Supplement: Supplementary file 1 — Supplementary Material 1 [file 12937_2024_930_MOESM1_ESM.docx]

**Supplementary Table 1** Comparisons of demographic characteristics between 121 ASD children recruited for this study and 93 ASD children recruited from July 2018 to May 2019

| **Variables** | **ASD (*n* = 121)** | **ASD (*n* = 93)** | ***p*** | **Effect size** |
| --- | --- | --- | --- | --- |
| **Child characteristics** |  |  |  |  |
| Age, median (IQR), y | 6 (4, 7) | 7 (6, 8) | NA | NA |
| Sex, n (%) |  |  |  |  |
| Male | 98 (81) | 79 (84.9) | 0.448 | 0.052 |
| Female | 23 (19) | 14 (15.1) |  |  |
| ASD symptom severity^a^, n (%) |  |  |  |  |
| Mild-to-moderate | 98 (81) | 64 (68.8) | 0.053 | 0.141 |
| Severe | 23 (19) | 29 (31.2) |  |  |
| Intellectual functioning^b^, n (%) |  |  |  |  |
| Normal and Borderline | 69 (57) | 51 (54.8) | 0.749 | 0.022 |
| Abnormal | 52 (43) | 42 (45.2) |  |  |
| **Maternal Characteristics** |  |  |  |  |
| Educational attainment, n (%) |  |  |  |  |
| College degree or below | 67 (55.4) | 48 (51.6) | 0.585 | 0.037 |
| Undergraduate degree or higher | 54 (44.6) | 45 (48.4) |  |  |
| **Family characteristics** |  |  |  |  |
| Monthly per-capita income, n (%) |  |  |  |  |
| ≤8,000 RMB | 86 (71.1) | 58 (62.4) | 0.178 | 0.092 |
| >8,000 RMB | 35 (28.9) | 35 (37.6) |  |  |

^a^ Evaluated by the Childhood Autism Rating Scale (CARS). ^b^ Evaluated by the Wechsler Intelligence Scale for Children Forth Edition (WISC-IV) or Gesell developmental Schedules (GDS). Effect size = effect size φ or Cramer-V for *χ^2^* tests. ASD: autism spectrum disorder, IQR: [interquartile range](javascript:;), RMB: Ren Min Bi (Chinese currency), NA: not applicable

| **Food items** | **Recommended daily food intake^c^** | | |
| --- | --- | --- | --- |
|  | **2–3 years old** | **4–5 years old** | **6–10 years old** |
| Cereals/potatoes^a^ | 75–125 | 100–150 | 175–250 |
| Vegetables^a^ | 100–200 | 150–300 | 300 |
| Fruit^a^ | 100–200 | 150–250 | 150–200 |
| Livestock/poultry meat^a^ | 35–60 | 30–55 | 40 |
| Fish/shrimp^a^ | 15 | 20 | 40 |
| Eggs^a^ | 50 | 50 | 25–40 |
| Soybeans^a^ | 5–15 | 15–20 | 15 |
| Nuts^a^ | -- | moderate amounts | 7 |
| Dairy^b^ | 350–500 | 350–500 | 300 |
| Cooking oil^a^ | 10–20 | 20–25 | 20–25 |
| Salt^a^ | <2 | <3 | <4 |
| Drinking water^b^ | 600–700 | 700–800 | 800–1000 |

**Supplementary Table 2** Recommended daily food intake for children aged 2 to 10 years old

^a^ g/day. ^b^ ml/day. ^c^ Recommendations from the Dietary Guidelines for Chinese Residents (2022) and Chinese Food Pagoda

**Supplementary Table 3** Scoring method of Dietary Balance Index (DBI) for children aged 2–3 years old

| **Components** | **Score range** | **Subgroups** | **Score range** | **Scoring method** |
| --- | --- | --- | --- | --- |
| Cereals and potatoes | (–12)–(12) | Cereals/potatoes | (–12)–(12) | 0g = –12, 75–125g = 0, >200g = 12  The score increased by 1 with the intake amount increased by 7g |
| Vegetables and fruit | (–12)–(0) | Vegetables | (–6)–(0) | 0g = –6, 80–99g = –1, ≥100g = 0  The score increased by 1 with the intake amount increased by 20g |
|  |  | Fruit | (–6)–(0) | 0g = –6, 80–99g = –1, ≥100g = 0  The score increased by 1 with the intake amount increased by 20g |
| Animal food | (–12)–(8) | Livestock/poultry meat | (–4)–(4) | <5g = –4  5–14g = –3  15–24g = –2  25–34g = –1  35–60g =0  61–70g = 1  71–80g = 2  81–90g = 3  >90g = 4 |
|  |  | Fish/shrimp | (–4)–(0) | 0g = –4  1–4g = –3  5–9g = –2  10–14g = –1  ≥15g = 0 |
|  |  | Eggs | (–4)–(4) | <5g = –4  5–19g = –3  20–34g = –2  35–49g = –1  50g = 0  51–65g = 1  66–80g = 2  81–95g = 3  >95g = 4 |
| Soybeans and dairy | (–12)–(0) | Soybeans | (–6)–(0) | 0g = –6, ≥5g = 0  The score increased by 1 with the intake amount increased by 1g |
|  |  | Dairy | (–6)–(0) | 0g = –6, 280–349g = –1, ≥350g = 0  The score increased by 1 with the intake amount increased by 70g |
| Empty energy food | 0–6 | Cooking oil | 0–6 | ≤20g = 0, 21–25g = 1, >45g = 6  The score increased by 1 with the intake amount increased by 5g |
| Condiments | 0–6 | Salt | 0–6 | <2g = 0, 2–3g = 1, ≥12g = 6  The score increased by 1 with the intake amount increased by 2g |
| Food variety | (–12)–(0) | Food variety | (–12)–(0) | ≥12 kinds of food = 0, the score is 0 when daily consumption of each food group reaches or exceeds 25g (soybeans 5g), otherwise score is –1 |
| Drinking water | (–12)–(0) | Drinking water | (–12)–(0) | <100ml = –12, ≥600ml = 0  The score decreased by 1 with the intake amount decreased by 46ml |

The DBI measured the intakes of the food groups including cereals/potatoes, vegetables, fruit, livestock/poultry meat, fish/shrimp, eggs, soybeans, dairy, cooking oil, salt, and drinking water. According to the Chinese Food Pagoda, cereals/potatoes, livestock/poultry meat, and eggs should be consumed in moderation, and thus a negative, zero, and positive score were obtained to signify inadequate, appropriate, and excessive intake, respectively. Sufficient vegetables, fruit, fish/shrimp, soybeans, dairy, and drinking water should be consumed, and thus a negative and zero score was obtained to characterize inadequate and appropriate intake. Proper cooking oil and salt should be consumed, and therefore a zero and a positive score was obtained to indicate appropriate and excessive intake. The definition of food variety scores included 12 kinds of food groups, namely rice and its products, wheat and its products, coarse grains and potatoes, dark-colored vegetables, light-colored vegetables, fruit, livestock meat, poultry meat, fish/shrimp, eggs, soybeans, and dairy. The minimum for each food group limit is 25g except 5g for soybeans. The score is 0 when the daily consumption of each food group reaches or exceeds 25g (soybeans 5g), otherwise score is –1, and the total food variety scores range from –12 to 0. The DBI consists of three main evaluation indexes namely high bound score (HBS), low bound score (LBS), and diet quality distance (DQD). HBS is the sum of all positive values with higher scores reflecting the degree of excessive consumption. LBS is the sum of absolute values of negative scores with higher scores indicating the degree of insufficient consumption. DQD is the sum of HBS and LBS with higher scores representing the degree of unbalanced consumption. The above index scores <20% “more appropriate”, 20–40% “low”, 40–60% “moderate”, >60% “high”, and a score of 0 “appropriate”

**Supplementary Table 4** Scoring method of Dietary Balance Index (DBI) for children aged 4–5 years old

| **Components** | **Score range** | **Subgroups** | **Score range** | **Scoring method** |
| --- | --- | --- | --- | --- |
| Cereals and potatoes | (–12)–(12) | Cereals/potatoes | (–12)–(12) | 0g = –12, 100–150g = 0, >250g = 12  The score increased by 1 with the intake amount increased by 9g |
| Vegetables and fruit | (–12)–(0) | Vegetables | (–6)–(0) | 0g = –6, 120–149g = –1, ≥150g = 0  The score increased by 1 with the intake amount increased by 30g |
|  |  | Fruit | (–6)–(0) | 0g = –6, 120–149g = –1, ≥150g = 0  The score increased by 1 with the intake amount increased by 30g |
| Animal food | (–12)–(8) | Livestock/poultry meat | (–4)–(4) | 0g = –4  1–9g = –3  10–19g = –2  20–29g = –1  30–55g =0  56–65g = 1  66–75g = 2  76–85g = 3  >85g = 4 |
|  |  | Fish/shrimp | (–4)–(0) | <5g = –4  5–9g = –3  10–14g = –2  15–19g = –1  ≥20g = 0 |
|  |  | Eggs | (–4)–(4) | <5g = –4  5–19g = –3  20–34g = –2  35–49g = –1  50g = 0  51–65g = 1  66–80g = 2  81–95g = 3  >95g = 4 |
| Soybeans and dairy | (–12)–(0) | Soybeans | (–6)–(0) | 0g = –6, ≥15g = 0  The score increased by 1 with the intake amount increased by 3g |
|  |  | Dairy | (–6)–(0) | 0g = –6, 280–349g = –1, ≥350g = 0  The score increased by 1 with the intake amount increased by 70g |
| Empty energy food | 0–6 | Cooking oil | 0–6 | ≤25g = 0, 26–30g = 1, >50g = 6  The score increased by 1 with the intake amount increased by 5g |
| Condiments | 0–6 | Salt | 0–6 | <3g = 0, 3–4g = 1, ≥13g = 6  The score increased by 1 with the intake amount increased by 2g |
| Food variety | (–12)–(0) | Food variety | (–12)–(0) | ≥12 kinds of food = 0, the score is 0 when daily consumption of each food group reaches or exceeds 25g (soybeans 5g), otherwise score is –1 |
| Drinking water | (–12)–(0) | Drinking water | (–12)–(0) | <100ml = –12, ≥700ml = 0  The score decreased by 1 with the intake amount decreased by 55ml |

**Supplementary Table 5** Scoring method of Dietary Balance Index (DBI) for children aged 6–10 years old

| **Components** | **Score range** | **Subgroups** | **Score range** | **Scoring method** |
| --- | --- | --- | --- | --- |
| Cereals and potatoes | (–12)–(12) | Cereals/potatoes | (–12)–(12) | 0g = –12, 175–250g = 0, >425g = 12  The score increased by 1 with the intake amount increased by 16g |
| Vegetables and fruit | (–12)–(0) | Vegetables | (–6)–(0) | 0g = –6, 240–299g = –1, ≥300g = 0  The score increased by 1 with the intake amount increased by 60g |
|  |  | Fruit | (–6)–(0) | 0g = –6, 120–149g = –1, ≥150g = 0  The score increased by 1 with the intake amount increased by 30g |
| Animal food | (–12)–(8) | Livestock/poultry meat | (–4)–(4) | <10g = –4  10–19g = –3  20–29g = –2  30–39g = –1  40g =0  41–50g = 1  51–60g = 2  61–70g = 3  >70g = 4 |
|  |  | Fish/shrimp | (–4)–(0) | <10g = –4  10–19g = –3  20–29g = –2  30–39g = –1  ≥40g = 0 |
|  |  | Eggs | (–4)–(4) | 0g = –4  1–8g = –3  9–16g = –2  17–24g = –1  25–40g = 0  41–48g = 1  49–56g = 2  57–64g = 3  >64g = 4 |
| Soybeans, nuts and dairy | (–12)–(0) | Soybeans and nuts | (–6)–(0) | 0g = –6, ≥25g = 0  The score increased by 1 with the intake amount increased by 5g |
|  |  | Dairy | (–6)–(0) | 0g = –6, 240–299g = –1, ≥300g = 0  The score increased by 1 with the intake amount increased by 60g |
| Empty energy food | 0–6 | Cooking oil | 0–6 | ≤25g = 0, 26–30g = 1, >50g = 6  The score increased by 1 with the intake amount increased by 5g |
| Condiments | 0–6 | Salt | 0–6 | <4g = 0, 4–5g = 1, ≥14g = 6  The score increased by 1 with the intake amount increased by 2g |
| Food variety | (–12)–(0) | Food variety | (–12)–(0) | ≥12 kinds of food = 0, the score is 0 when daily consumption of each food group reaches or exceeds 25g (soybeans and nuts 5g), otherwise score is –1 |
| Drinking water | (–12)–(0) | Drinking water | (–12)–(0) | <100ml = –12, ≥800ml = 0  The score decreased by 1 with the intake amount decreased by 64ml |

**Supplementary Table 6** The crude and adjusted associations between autism-linked traits and poorer diets and gastrointestinal symptoms in children with ASD (*n* = 121)

| **Autism-linked traits** | **Daily vegetable intake^a^** | | | |  | **Daily fruit intake^a^** | | | | |  | **Daily food variety^b^** | | |
| --- | --- | --- | --- | --- | --- | --- | --- | --- | --- | --- | --- | --- | --- | --- |
|  | **Crude *p*** | **Adjusted β coefficient**  **(95% CI)** | **Adjusted *p*** | |  | **Crude *p*** | **Adjusted β coefficient (95% CI)** | | | **Adjusted *p*** |  | **Crude *p*** | **Adjusted β coefficient (95% CI)** | **Adjusted *p*** |
| ASD symptom severity | 0.584 | 2.350 (–2.852 to 7.551) | 0.373 | |  | 0.533 | –1.546 (–5.827 to 2.736) | | | 0.476 |  | 0.163 | –0.082 (–0.174 to 0.011) | 0.084 |
| Stereotypic behavior | 0.212 | –6.470 (–14.454 to 1.515) | 0.111 | |  | 0.256 | –3.322 (–9.943 to 3.299) | | | 0.322 |  | 0.14 | –0.093 (–0.236 to 0.049) | 0.198 |
| Self-injurious behavior | 0.209 | –8.516 (–21.037 to 4.005) | 0.180 | |  | 0.125 | –6.574 (–16.891 to 3.743) | | | 0.209 |  | 0.001 | –0.357 (–0.571 to –0.143) | 0.001 |
| Compulsive behavior | 0.007 | –14.144 (–21.703 to –6.584) | <0.001 | |  | 0.572 | –1.884 (–8.491 to 4.723) | | | 0.573 |  | 0.053 | –0.146 (–0.287 to –0.006) | 0.041 |
| Ritualistic behavior | 0.017 | –10.445 (–18.757 to –2.132) | 0.014 | |  | 0.957 | 0.647 (–6.394 to 7.687) | | | 0.856 |  | 0.371 | –0.064 (–0.216 to 0.087) | 0.401 |
| Sameness behavior | 0.029 | –6.235 (–11.801 to –0.669) | 0.028 | |  | 0.6 | –0.827 (–5.512 to 3.858) | | | 0.727 |  | 0.36 | –0.049 (–0.150 to 0.052) | 0.342 |
| Restricted interests | 0.343 | –1.901 (–13.162 to 9.360) | 0.738 | |  | 0.571 | –0.990 (–10.262 to 8.282) | | | 0.833 |  | 0.13 | –0.167 (–0.365 to 0.030) | 0.096 |
| RBS-R total scores | 0.012 | –2.766 (–4.651 to –0.882) | 0.004 | |  | 0.366 | –0.553 (–2.162 to 1.056) | | | 0.497 |  | 0.042 | –0.037 (–0.071 to –0.003) | 0.035 |
| Taste/smell sensitivity | 0.05 | 4.941 (–0.898 to 10.780) | 0.096 | |  | 0.006 | 6.127 (1.403 to 10.851) | | | 0.012 |  | 0.001 | 0.151 (0.050 to 0.252) | 0.004 |
| Tactile sensitivity | 0.933 | –0.989 (–7.665 to 5.687) | 0.770 | |  | 0.205 | 3.621 (–1.831 to 9.073) | | | 0.191 |  | 0.93 | –0.015 (–0.134 to 0.103) | 0.796 |
| Movement sensitivity | 0.559 | 1.142 (–8.412 to 10.696) | 0.813 | |  | 0.618 | –0.661 (–8.526 to 7.204) | | | 0.868 |  | 0.933 | 0.021 (–0.149 to 0.191) | 0.806 |
| Underresponsive/seeks sensation | 0.655 | 1.158 (–3.765 to 6.082) | 0.642 | |  | 0.126 | 2.832 (–1.187 to 6.851) | | | 0.165 |  | 0.341 | 0.042 (–0.045 to 0.130) | 0.337 |
| Auditory filtering | 0.409 | 1.963 (–3.986 to 7.911) | 0.514 | |  | 0.026 | 5.448 (0.656 to 10.239) | | | 0.026 |  | 0.722 | 0.006 (–0.100 to 0.112) | 0.906 |
| Low energy/weak | 0.444 | –0.146 (–4.997 to 4.706) | 0.953 | |  | 0.01 | 4.482 (0.584 to 8.381) | | | 0.025 |  | 0.767 | 0.010 (–0.076 to 0.097) | 0.812 |
| Visual/auditory sensitivity | 0.526 | 2.123 (–4.288 to 8.533) | 0.513 | |  | 0.822 | 0.213 (–5.074 to 5.500) | | | 0.936 |  | 0.671 | 0.024 (–0.090 to 0.139) | 0.672 |
| SSP total scores | 0.23 | 0.507 (–0.836 to 1.850) | 0.456 | |  | 0.012 | 1.295 (0.216 to 2.375) | | | 0.019 |  | 0.217 | 0.013 (–0.011 to 0.037) | 0.283 |
| Food refusal | 0.607 | –1.623 (–11.622 to 8.376) | 0.748 | |  | 0.576 | –0.574 (–8.807 to 7.659) | | | 0.890 |  | 0.698 | 0.018 (–0.160 to 0.196) | 0.838 |
| Limited variety of foods | 0.006 | –5.970 (–10.604 to –1.337) | 0.012 | |  | 0.122 | –2.964 (–6.852 to 0.925) | | | 0.134 |  | 0.002 | –0.143 (–0.223 to –0.063) | 0.001 |
| Features of autism | 0.153 | –5.553 (–15.343 to 4.237) | 0.263 | |  | 0.242 | –4.707 (–12.762 to 3.348) | | | 0.249 |  | 0.263 | –0.085 (–0.259 to 0.090 | 0.337 |
| BAMBI total scores | 0.018 | –3.744 (–7.006 to –0.482) | 0.025 | |  | 0.122 | 0.137 | | | 0.137 |  | 0.016 | –0.078 (–0.135 to –0.020) | 0.009 |
|  | **Inadequate dietary intake^c^** | | | | | |  | **Unbalanced dietary intake^c^** | | | | | | |
|  | **Crude *p*** | **Adjusted β coefficient (95% CI)** | | **Adjusted *p*** | | |  | **Crude *p*** | **Adjusted β coefficient (95% CI)** | | | | **Adjusted *p*** | |
| ASD symptom severity | 0.046 | 0.470 (0.053 to 0.887) | | 0.028 | | |  | 0.034 | 0.531 (0.126 to 0.937) | | | | 0.011 | |
| Stereotypic behavior | 0.882 | –0.034 (–0.682 to 0.614) | | 0.917 | | |  | 0.872 | 0.159 (–0.470 to 0.789) | | | | 0.617 | |
| Self-injurious behavior | 0.066 | 1.098 (0.108 to 2.088) | | 0.030 | | |  | 0.068 | 0.974 (0.007 to 1.940) | | | | 0.048 | |
| Compulsive behavior | 0.416 | 0.419 (–0.220 to 1.059) | | 0.196 | | |  | 0.564 | 0.325 (–0.229 to 0.949) | | | | 0.304 | |
| Ritualistic behavior | 0.315 | 0.396 (–0.286 to 1.077) | | 0.252 | | |  | 0.099 | 0.495 (–0.165 to 1.156) | | | | 0.140 | |
| Sameness behavior | 0.965 | 0.138 (–0.318 to 0.594) | | 0.549 | | |  | 0.563 | 0.196 (–0.246 to 0.639) | | | | 0.381 | |
| Restricted interests | 0.535 | 0.596 (–0.300 to 1.492) | | 0.190 | | |  | 0.441 | 0.560 (–0.312 to 1.432) | | | | 0.205 | |
| RBS-R total scores | 0.459 | 0.104 (–0.052 to 0.260) | | 0.189 | | |  | 0.273 | 0.119 (–0.032 to 0.270) | | | | 0.123 | |
| Taste/smell sensitivity | 0.008 | –0.570 (–1.031 to –0.108) | | 0.016 | | |  | 0.011 | –0.515 (–0.965 to –0.064) | | | | 0.026 | |
| Tactile sensitivity | 0.531 | 0.178 (–0.356 to 0.713) | | 0.510 | | |  | 0.8 | 0.149 (–0.371 to 0.670) | | | | 0.570 | |
| Movement sensitivity | 0.665 | 0.066 (–0.700 to 0.832) | | 0.864 | | |  | 0.757 | 0.081 (–0.664 to 0.826) | | | | 0.830 | |
| Underresponsive/seeks sensation | 0.518 | –0.094 (–0.488 to 0.301) | | 0.639 | | |  | 0.115 | –0.304 (–0.684 to 0.076) | | | | 0.115 | |
| Auditory filtering | 0.623 | 0.181 (–0.295 to 0.658) | | 0.453 | | |  | 0.468 | –0.142 (–0.607 to 0.322) | | | | 0.544 | |
| Low energy/weak | 0.577 | –0.117 (–0.506 to 0.271) | | 0.550 | | |  | 0.123 | –0.274 (–0.648 to 0.101) | | | | 0.151 | |
| Visual/auditory sensitivity | 0.875 | 0.099 (–0.416 to 0.613) | | 0.705 | | |  | 0.845 | –0.004 (–0.505 to 0.497) | | | | 0.988 | |
| SSP total scores | 0.557 | –0.023 (–0.131 to 0.084) | | 0.668 | | |  | 0.143 | –0.070 (–0.174 to 0.034) | | | | 0.184 | |
| Food refusal | 0.832 | 0.132 (–0.669 to 0.934) | | 0.744 | | |  | 0.321 | 0.624 (–0.147 to 1.395) | | | | 0.111 | |
| Limited variety of foods | 0.011 | 0.671 (0.311 to 1.031) | | <0.001 | | |  | 0.01 | 0.662 (0.312 to 1.012) | | | | <0.001 | |
| Features of autism | 0.353 | 0.553 (–0.230 to 1.335) | | 0.164 | | |  | 0.684 | 0.274 (–0.492 to 1.041) | | | | 0.479 | |
| BAMBI total scores | 0.075 | 0.407 (0.151 to 0.663) | | 0.002 | | |  | 0.036 | 0.425 (0.178 to 0.673) | | | | 0.001 | |
|  | **Constipation^d^** | | | | | |  | **Gastrointestinal symptoms^d^** | | | | | | |
|  | **Crude *p*** | **Adjusted β coefficient (95% CI)** | | **Adjusted *p*** | | |  | **Crude *p*** | **Adjusted β coefficient (95% CI)** | | | | **Adjusted *p*** | |
| ASD symptom severity | 0.774 | 0.000 (–0.028 to 0.029) | | 0.974 | | |  | 0.667 | 0.012 (–0.042 to 0.066) | | | | 0.665 | |
| Stereotypic behavior | 0.573 | –0.007 (–0.051 to 0.038) | | 0.770 | | |  | 0.264 | 0.042 (–0.042 to 0.125) | | | | 0.323 | |
| Self-injurious behavior | 0.831 | –0.003 (–0.073 to 0.066) | | 0.922 | | |  | 0.155 | 0.045 (–0.085 to 0.175) | | | | 0.493 | |
| Compulsive behavior | 0.095 | –0.031 (–0.075 to 0.013) | | 0.162 | | |  | 0.056 | –0.051 (–0.186 to 0.025) | | | | 0.091 | |
| Ritualistic behavior | 0.383 | –0.028 (–0.074 to 0.019) | | 0.240 | | |  | 0.268 | –0.066 (–0.154 to 0.021) | | | | 0.136 | |
| Sameness behavior | 0.31 | –0.018 (–0.049 to 0.013) | | 0.257 | | |  | 0.48 | –0.038 (–0.097 to 0.020) | | | | 0.197 | |
| Restricted interests | 0.515 | –0.032 (–0.094 to 0.029) | | 0.299 | | |  | 0.638 | –0.051 (–0.167 to 0.065) | | | | 0.388 | |
| RBS-R total scores | 0.232 | –0.007 (–0.018 to 0.004) | | 0.203 | | |  | 0.601 | –0.012 (–0.032 to 0.008) | | | | 0.229 | |
| Taste/smell sensitivity | 0.996 | 0.001 (–0.031 to 0.034) | | 0.928 | | |  | 0.294 | –0.027 (–0.088 to 0.033) | | | | 0.374 | |
| Tactile sensitivity | 0.902 | 0.003 (–0.034 to 0.039) | | 0.884 | | |  | 0.123 | –0.026 (–0.095 to 0.042) | | | | 0.450 | |
| Movement sensitivity | 0.181 | –0.019 (–0.071 to 0.034) | | 0.481 | | |  | 0.148 | –0.049 (–0.147 to 0.049) | | | | 0.324 | |
| Underresponsive/seeks sensation | 0.389 | 0.012 (–0.015 to 0.039) | | 0.370 | | |  | 0.45 | 0.016 (–0.035 to 0.066) | | | | 0.546 | |
| Auditory filtering | 0.592 | 0.009 (–0.023 to 0.042) | | 0.574 | | |  | 0.911 | 0.005 (–0.057 to 0.066) | | | | 0.879 | |
| Low energy/weak | 0.649 | –0.002 (–0.028 to 0.025) | | 0.907 | | |  | 0.089 | –0.020 (–0.070 to 0.030) | | | | 0.420 | |
| Visual/auditory sensitivity | 0.326 | 0.014 (–0.021 to 0.049) | | 0.424 | | |  | 0.798 | –0.007 (–0.073 to 0.060) | | | | 0.844 | |
| SSP total scores | 0.824 | 0.002 (–0.006 to 0.009) | | 0.648 | | |  | 0.292 | –0.004 (–0.018 to 0.010) | | | | 0.576 | |
| Food refusal | 0.854 | –0.009 (–0.064 to 0.045) | | 0.738 | | |  | 0.669 | –0.032 (–0.135 to 0.071) | | | | 0.542 | |
| Limited variety of foods | 0.404 | 0.010 (–0.016 to 0.036) | | 0.433 | | |  | 0.551 | 0.013 (–0.036 to 0.063) | | | | 0.590 | |
| Features of autism | 0.763 | –0.018 (–0.071 to 0.036) | | 0.517 | | |  | 0.572 | –0.014 (–0.116 to 0.087) | | | | 0.778 | |
| BAMBI total scores | 0.723 | 0.002 (–0.016 to 0.020) | | 0.829 | | |  | 0.477 | 0.001 (–0.033 to 0.036) | | | | 0.938 | |
| Daily vegetable intake | 0.75 | 0.000 (–0.001 to 0.001) | | 0.943 | | |  | 0.412 | 0.000 (–0.002 to 0.002) | | | | 0.969 | |
| Daily fruit intake | 0.278 | –0.001 (–0.002 to 0.001) | | 0.309 | | |  | 0.114 | –0.002 (–0.004 to 0.001) | | | | 0.162 | |
| Daily food variety | 0.411 | –0.029 (–0.088 to 0.030) | | 0.337 | | |  | 0.184 | –0.074 (–0.185 to 0.038) | | | | 0.192 | |
| Inadequate dietary intake | 0.218 | 0.010 (–0.003 to 0.023) | | 0.120 | | |  | 0.121 | 0.024 (0.000 to 0.049) | | | | 0.052 | |
| Unbalanced dietary intake | 0.04 | 0.013 (0.001 to 0.026) | | 0.040 | | |  | 0.027 | 0.027 (0.002 to 0.052) | | | | 0.032 | |

^a^ g/day. ^b^ Food variety scores ranged from –12 to 0, with lower scores representing less variety of food. ^c^ Evaluated using the low bound score (LBS)/diet quality distance (DQD), with higher scores representing a higher degree of inadequate/unbalanced diets. ^d^ Constipation and gastrointestinal symptoms were evaluated by subscale and total scores of the 6-item gastrointestinal severity index (6-GSI). ASD symptom severity was evaluated by the Childhood Autism Rating Scale (CARS). Restricted repetitive behaviors, sensory profiles, and mealtime behaviors were evaluated by subscale and total scores of repetitive behavior scale-revised (RBS-R), short sensory profile (SSP), and brief autism mealtime behavior inventory (BAMBI), respectively. Adjusted for child’s age, sex, intellectual functioning, birth mode, average daily sleep duration, average daily SB time, average daily MVPA time, average daily walking time, maternal educational attainment, monthly per-capita income, and parenting behavior. ASD: autism spectrum disorder, CI: confidence interval, SB: sedentary behavior, MVPA: moderate-to-vigorous physical activity

**Supplementary Table 7** The adjusted associations between autism-linked traits and poorer diets in children with ASD (*n* = 121)

| Autism-linked traits | **Daily vegetable intake^a^** | | |  | **Daily fruit intake^a^** | | | |  | **Daily food variety^b^** | | | |
| --- | --- | --- | --- | --- | --- | --- | --- | --- | --- | --- | --- | --- | --- |
|  | **Adjusted β coefficient (95% CI)** | ***p*** | **Effect size** |  | **Adjusted β coefficient (95% CI)** | | ***p*** | **Effect size** |  | **Adjusted β coefficient (95% CI)** | | ***p*** | **Effect size** |
| Self-injurious behavior |  |  | 0.277 |  |  | |  | 0.250 |  | –0.337 (–0.580 to –0.094) | | 0.007 | 0.312 |
| Compulsive behavior | –16.047 (–29.197 to –2.897) | 0.017 |  |  |  | |  |  |  | –0.132 (–0.346 to 0.082) | | 0.223 |  |
| Ritualistic behavior | –5.782 (–19.596 to 8.033) | 0.408 |  |  |  | |  |  |  |  | |  |  |
| Sameness behavior | –0.214 (–11.656 to 11.228) | 0.970 |  |  |  | |  |  |  |  | |  |  |
| RBS-R total scores | 2.063 (–3.712 to 7.839) | 0.480 |  |  |  | |  |  |  | 0.034 (–0.024 to 0.093) | | 0.247 |  |
| Taste/smell sensitivity |  |  |  |  | 5.798 (0.020 to 11.616) | | 0.049 |  |  | 0.000 (–0.132 to 0.132) | | 0.995 |  |
| Auditory filtering |  |  |  |  | 5.055 (–1.161 to 11.270) | | 0.110 |  |  |  | |  |  |
| Low energy/weak |  |  |  |  | 4.159 (–1.139 to 9.456) | | 0.123 |  |  |  | |  |  |
| SSP total scores |  |  |  |  | –0.904 (–2.972 to 1.165) | | 0.388 |  |  |  | |  |  |
| Limited variety of foods | –3.039 (–13.271 to 7.193) | 0.557 |  |  |  | |  |  |  | –0.215 (–0.416 to –0.014) | | 0.036 |  |
| BAMBI total scores | –0.746 (–7.768 to 6.275) | 0.833 |  |  |  | |  |  |  | 0.067 (–0.058 to 0.192) | | 0.289 |  |
|  | **Inadequate dietary intake^c^** | | | | |  | **Unbalanced dietary intake^c^** | | | | | | |
|  | **Adjusted β coefficient (95% CI)** | ***p*** | | | **Effect size** |  | **Adjusted β coefficient (95% CI)** | | | | ***p*** | **Effect size** | |
| ASD symptom severity | 0.554 (0.117 to 0.990) | 0.014 | | | 0.305 |  | 0.609 (0.184 to 1.035) | | | | 0.005 | 0.350 | |
| Self-injurious behavior | 0.794 (–0.229 to 1.817) | 0.127 | | |  |  | 0.666 (–0.331 to 1.663) | | | | 0.188 |  |  |
| Taste/smell sensitivity | 0.048 (–0.555 to 0.651) | 0.874 | | |  |  | 0.085 (–0.502 to 0.672) | | | | 0.774 |  |  |
| Limited variety of foods | 0.770 (0.138 to 1.678) | 0.004 | | |  |  | 0.639 (0.245 to 1.524) | | | | 0.010 |  |  |
| BAMBI total scores | –0.109 (–0.683 to 0.465) | 0.708 | | |  |  | 0.009 (–0.550 to 0.568) | | | | 0.974 |  |  |

^a^ g/day. ^b^ Food variety scores ranged from –12 to 0, with lower scores representing less variety of food. ^c^ Evaluated using the low bound score (LBS)/diet quality distance (DQD), with higher scores representing a higher degree of inadequate/unbalanced diets. ASD symptom severity was evaluated by the Childhood Autism Rating Scale (CARS). Restricted repetitive behaviors, sensory profiles, and mealtime behaviors were evaluated by subscale and total scores of repetitive behavior scale-revised (RBS-R), short sensory profile (SSP), and brief autism mealtime behavior inventory (BAMBI), respectively. Adjusted for child’s age, sex, intellectual functioning, birth mode, average daily sleep duration, average daily SB time, average daily MVPA time, average daily walking time, maternal educational attainment, monthly per-capita income, and parenting behavior. Effect size = Cohen's f^2^ for linear models. ASD: autism spectrum disorder, CI: confidence interval, SB: sedentary behavior, MVPA: moderate-to-vigorous physical activity

**Supplementary Table 8** Covariates definitions

| **Variables** | **Definition** | **Source** | **Type** | **Analysis format** |
| --- | --- | --- | --- | --- |
| Age | Years | Demographic questionnaire | Continuous |  |
| Sex | Sex | Demographic questionnaire | Dichotomous | Male  Female |
| Intellectual functioning | Score | Wechsler Intelligence Scale for Children Forth Edition (WISC-IV): applied to children aged 6 to 10 years [1]  Gesell developmental Schedules (GDS): applied to children aged < 6 years [2] | Categorical | WISC-IV:  Normal (score≥80)  Borderline (80>score≥70)  Abnormal (score<70)  GDS:  Normal (score>85)  Borderline (85≥score≥76)  Abnormal (score<76) |
| Birth mode | Birth mode | Demographic questionnaire | Categorical | Vaginal delivery  Elective caeserean section  Emergency caeserean section |
| Birth order | Birth order | Demographic questionnaire | Dichotomous | 1  >1 |
| Average daily sleep duration | Hours/day | Demographic questionnaire:  Sleep duration during the night and day | Continuous |  |
| Average daily SB time | Hours/day | Assessment questionnaire:  International Physical Activity Questionnaire Short-Form (IPAQ-SF) [3, 4] | Continuous |  |
| Average daily moderate-to-vigorous physical activity time | Hours/day | Assessment questionnaire:  International Physical Activity Questionnaire Short-Form (IPAQ-SF) | Continuous |  |
| Average daily walking time | Hours/day | Assessment questionnaire:  International Physical Activity Questionnaire Short-Form (IPAQ-SF) | Continuous |  |
| Maternal educational attainment | Educational attainment | Demographic questionnaire | Dichotomous | College degree or below  Undergraduate degree or higher |
| Gestational diabetes mellitus | Doctor-diagnosed | 75g oral glucose tolerance test (OGTT) administered at 24 to 28 weeks of gestation [5] | Dichotomous | Yes  No |
| Maternal obesity | Doctor-diagnosed | Body mass index (BMI) ≥ 30kg/m [6, 7] | Dichotomous | Yes  No |
| Monthly per-capita income | Renminbi | Demographic questionnaire | Dichotomous | ≤ 8,000  > 8,000 |
| Parenting behavior | Score | Assessment questionnaire:  Parent Behavior Inventory (PBI) [8] | Dichotomous | Support/engagement  Opposition/defiance |

**References**

1. Kaufman AS, Flanagan DP, Alfonso VC, Mascolo JT. Test Review: Wechsler Intelligence Scale for Children, Fourth Edition (WISC-IV). Journal of Psychoeducational Assessment. 2006;24(3):278–95.

2. Dror R, Malinger G, Ben-Sira L, Lev D, Pick CG, Lerman-Sagie T. Developmental outcome of children with enlargement of the cisterna magna identified in utero. J Child Neurol. 2009;24(12):1486–92.

3. Macfarlane DJ, Lee CCY, Ho EYK, Chan KL, Chan DTS. Reliability and validity of the Chinese version of IPAQ (short, last 7 days). J Sci Med Sport. 2007; 10(1):45–51.

4. Craig CL, Marshall AL, Sjöström M, Bauman AE, Booth ML, Ainsworth BE, et al. International physical activity questionnaire: 12-country reliability and validity. Med Sci Sports Exerc. 2003;35(8):1381–95.

5. International Association of Diabetes and Pregnancy Study Groups Consensus Panel, Metzger BE, Gabbe SG, Persson B, Buchanan TA, Catalano PA, Damm P, et al. International association of diabetes and pregnancy study groups recommendations on the diagnosis and classification of hyperglycemia in pregnancy. Diabetes care. 2010;33(3):676–82.

6. Xian DM, Xu XM, Zhong JX, Liu N. Summary of best evidences on obesity management in pregnant women. Journal of Nursing Science. 2021;36(16):88–91.

7. Tong L, Kalish BT. The impact of maternal obesity on childhood neurodevelopment. J Perinatol. 2021;41(5):928–39.

8. Lovejoy MC, Weis R, O'Hare E, Rubin EC. Development and initial validation of the Parent Behavior Inventory. Psychological assessment. 1999;11(4):534–45.

**Supplementary methods**

**Assessment of gastrointestinal symptoms**

A shortened version of the 6-item Gastrointestinal Severity Index (6-GSI) questionnaire was intended to assess the severity of gastrointestinal symptoms among patients with ASD in the present study. In a 2001 study, it was described firstly and employed as an instrument to measure aspects of gastrointestinal symptoms in ASD research [9]. The 6-GSI is composed of six symptoms including constipation, diarrhea, stool consistency, stool smell, flatulence, and abdominal pain. Each symptom is rated on a three-point Likert-type scale ranging from 0 to 2 with a higher score signifying more severe gastrointestinal symptoms [10]. The interrater reliability (intraclass correlation (ICC)) of the gastrointestinal total scores was found to be high at 0.95 (95% confidence interval (CI): 0.87–0.98) [10].

**Measures of Autism-linked traits**

**ASD symptom severity**

The Childhood Autism Rating Scale (CARS) is a behaviorally based clinical scale derived from interaction and observation [11]. It contains 15 items rated from 1 (age appropriate) to 4 (severely autistic), covering language and communication skills, response to sensory information, and socio-emotional and interactional skills. CARS had demonstrated a high degree of internal consistency with a Cronbach’s alpha of 0.92

and high validity with a sensitivity of 87.5% and specificity 90% [12]. The CARS was applied in this study to distinguish between mild-to-moderate and severe ASD. According to the manual, a CARS score between 30 and 36.5 is indicative of mild-to-moderate ASD, and a score between 37 and 60 represents severe ASD [13].

**Restricted repetitive behaviors**

Repetitive Behavior Scale-Revised (RBS-R) is a parent or caregiver-report scale for capturing the breadth of restricted repetitive behaviors observed in individuals with ASD. The RBS-R has sound psychometric characteristics. Crohnbach’s alphas for all of the subscales were satisfactorily high (between 0.78 to 0.91, mean = 0.83) [14]. The 43 items of RBS-R have been conceptually grouped into 6 subscales that each represents the principal typologies of restricted repetitive behaviors: stereotypic behavior, self-injurious behavior, compulsive behavior, ritualistic behavior, sameness behavior, and restricted interests. Every item is rated on a 0-point (never) to 3-point (always) Likert-type scale; thus, higher scores reflect more severe stereotypical and repetitive behavior.

**Sensory profiles**

The short sensory profile (SSP), an abbreviate form of Dunn’s Sensory Profile, is one of the primary measures of sensory phenotyping among autistic children [15]. It has adequate internal consistency with Cronbach’s alpha coefficient ranging from 0.70 to 0.90 [16]. The validated Chinese version of SSP has been established by Xu et al. with good reliability and validity [17]. The test-retest reliability coefficient of the SSP is above 0.7, and the correlation coefficient between each of the subscales and the total SSP is above 0.5 [17]. The SSP consists of 38 items divided into 7 subscales, each corresponding to one of the sensory subtypes: tactile, taste/smell, movement, underresponsive/seeks sensation, visual/auditory sensitivity, auditory filtering, and low energy/weak. Each item is answered on a five-point Likert-type scale ranging from always (1) to never (5), with higher total and subscale scores indicating more typical performance while low scores emblematize heightened sensitivity in that sensory domain.

**Mealtime behaviors**

The Brief Autism Mealtime Behaviour Inventory (BAMBI) is a standardized and parent-reported measure of mealtime behaviors in ASD children with adequate reliability based on internal consistency scores (Cronbach’s alpha coefficient was 0.88) [18]. A test–retest reliability coefficient was calculated between the initial administration of the BAMBI and the second administration using the total score and was found to be significant with r of 0.87 [18]. For the criterion-related validity, there existed significant correlation between the BAMBI total frequency score and the Behavioral Pediatric Feeding Assessment Scale (BPFAS) child behavior frequency score with r of 0.77 [18]. The BAMBI contains 18 items ranked on a five-point Likert level ranging from 1 (never/rarely) to 5 (almost every meal) and is divided into three subscales that include limited variety, food refusal, and features of ASD. The items were summed to create total scores and subscale scores. Higher scores stand for a greater frequency of problematic behaviors.

**References**

9. Adams JB, Johansen LJ, Powell LD, Quig D, Rubin RA. Gastrointestinal flora and gastrointestinal status in children with autism--comparisons to typical children and correlation with autism severity. BMC Gastroenterol. 2011;11:22.

10. Thulasi V, Steer RA, Monteiro IM, Ming X. Overall severities of gastrointestinal symptoms in pediatric outpatients with and without autism spectrum disorder. Autism. 2019;23:524–30.

11. Kaat A, Lecavalier L. Childhood Autism Rating Scale. Encyclopedia of autism spectrum disorders. 2013;590–3.

12. Nordin V, Gillberg C, Nydén A. The Swedish version of the Childhood Autism Rating Scale in a clinical setting. J Autism Dev Disord. 1998;28:69–75.

13. Jiao Y, Chen R, Ke XY, Cheng L, Chu KK, Lu ZH, et al. Single nucleotide polymorphisms predict symptom severity of autism spectrum disorder. Journal of Autism and Developmental Disorders. 2012;42:971–83.

14. Lam KSL, Aman MG. The Repetitive Behavior Scale-Revised: independent validation in individuals with autism spectrum disorders. J Autism Dev Disord. 2007;37:855–66.

15. Rogers SJ, Hepburn S, Wehner E. Parent reports of sensory symptoms in toddlers with autism and those with other developmental disorders. J Autism Dev Disord. 2003;33:631–42.

16. Tomchek SD, Dunn W. Sensory processing in children with and without autism: a comparative study using the short sensory profile. Am J Occup Ther. 2007;61:190–200.

17. Zhou BR, Xu Q, Lu P, Yu X, Dunn W, Xu X. Reliability and validity analysis and clinical application of the Chinese version of Short Sensory Profile. China Conference Proceedings Database. 2016;238–9.

18. Lukens CT, Linscheid TR. Development and validation of an inventory to assess mealtime behavior problems in children with autism. J Autism Dev Disord. 2008;38:342–52.

**Supplementary Table 9** Comparison of dietary intake between children with ASD and age-matched TD children

| **Dietary intake** | **ASD (*n* = 121)** | **TD (*n* = 121)** | ***p*** | **Effect**  **Size** | **ASD (*n* = 48)** | **TD (*n* = 48)** | ***p*** | **Effect**  **Size** | **ASD (*n* = 73)** | **TD (*n* = 73)** | ***p*** | **Effect**  **size** |
| --- | --- | --- | --- | --- | --- | --- | --- | --- | --- | --- | --- | --- |
|  | **2–10 years old** | **2–10 years old** |  |  | **2–5 years old** | **2–5 years old** |  |  | **6–10 years old** | **6–10 years old** |  |  |
| Cereals/potatoes^a^, median (IQR) | 200 (150, 300) | 200 (150, 300) | 0.786 | 0.017 | 200 (150, 300) | 150 (150, 200) | 0.198 | 0.131 | 200 (150, 300) | 250 (150, 325) | 0.571 | 0.047 |
| Vegetables^a^, median (IQR) | 100 (35.7, 160.7) | 100 (82.4, 200) | 0.002 | 0.195 | 100 (35.7, 148.2) | 100 (71.4, 200) | 0.035 | 0.215 | 100 (39.3, 185.7) | 100 (85.7, 200) | 0.026 | 0.185 |
| Fruit^a^, median (IQR) | 50 (21.4, 100) | 75 (50, 142.9) | <0.001 | 0.231 | 50 (21.4, 112.5) | 71.4 (44.6, 147.5) | 0.049 | 0.201 | 50 (35.7, 100) | 75 (50, 142.9) | 0.002 | 0.253 |
| Livestock/poultry meat^a^,  median (IQR) | 100 (50, 200) | 100 (71.4, 200) | 0.015 | 0.157 | 100 (44.6, 192.9) | 100 (50, 105.4) | 0.985 | 0.002 | 100 (50, 200) | 150 (100, 200) | 0.001 | 0.265 |
| Fish/shrimp^a^, median (IQR) | 14.3 (0, 35.7) | 21.4 (14.3, 42.9) | 0.003 | 0.194 | 21.4 (0, 35.7) | 21.4 (7.1, 42.9) | 0.223 | 0.124 | 14.3 (5.7, 32.1) | 28.6 (14.3, 46.4) | 0.003 | 0.247 |
| Eggs^a^, median (IQR) | 35.7 (14.3, 50) | 35.7 (21.4, 50) | 0.380 | 0.056 | 35.7 (11.6, 50) | 28.6 (15.2, 41.1) | 0.275 | 0.112 | 35.7 (14.3, 50) | 42.9 (28.6, 51.8) | 0.030 | 0.180 |
| Soybeans^a^, median (IQR) | 7.1 (0, 16.1) | 10.7 (0.7, 21.4) | 0.104 | 0.105 | 4.6 (0, 19.6) | 12.5 (0, 21.4) | 0.153 | 0.146 | 7.1 (0, 16.1) | 7.1 (1.4, 21.4) | 0.382 | 0.072 |
| Dairy^b^, median (IQR) | 200 (107.1, 300) | 250 (200, 378.6) | 0.012 | 0.162 | 250 (150, 408.6) | 250 (183.9, 357.1) | 0.988 | 0.002 | 200 (85.7, 250) | 250 (200, 400) | 0.001 | 0.263 |
| Food variety^c^, mean ± SD | –5 (–6, –3) | –4 (–5, –2) | <0.001 | 0.300 | –5 (–7, –3) | –4 (–6, –2) | 0.015 | 0.248 | –5 (–6, –3.5) | –4 (–4.5, –2) | <0.001 | 0.333 |
| Drinking water^b^, median (IQR) | 750 (500, 1250) | 750 (500, 1000) | 0.431 | 0.051 | 750 (500, 1000) | 500 (500, 750) | 0.101 | 0.167 | 750 (500, 1250) | 750 (500, 1250) | 0.777 | 0.024 |
| HBS, median (IQR) | 8 (4, 13) | 6 (4, 12) | 0.281 | 0.069 | 11 (4, 13.8) | 6 (3.3, 12) | 0.029 | 0.223 | 7 (4, 12) | 7 (4, 11.5) | 0.620 | 0.041 |
| LBS, mean ± SD | –23.5 ± 9.8 | –17.7 ± 8.7 | <0.001 | 0.301 | –22 ± 12.2 | –16.9 ± 9.5 | 0.023 | 0.223 | –24.5 ± 7.7 | –18.3 ± 8.1 | <0.001 | 0.353 |
| DQD, mean ± SD | 32.1 ± 9.7 | 25.6 ± 8.9 | <0.001 | 0.323 | 31.4 ± 11.5 | 23.8 ± 9.8 | 0.001 | 0.326 | 32.6 ± 8.4 | 26.8 ± 8.1 | <0.001 | 0.329 |

^a^ g/day. ^b^ ml/day. ^c^ Food variety scores ranged from –12 to 0, with lower scores representing less variety of food. Excessive/inadequate/unbalanced dietary intake was evaluated using the high bound score (HBS), low bound score (LBS), and diet quality distance (DQD), respectively. Effect size = Z/√(N) for Mann–Whitney U-tests. ASD: autism spectrum disorder, TD: typically developing, IQR: [interquartile range](javascript:;), SD: standard deviation

**Supplementary Table 10** Factor loadings for food groups in three principal-component factors of children with ASD and age-matched TD children (*n* = 242)

| **Variables** | **ASD (*n* = 121)** | | |  | **TD (*n* = 121)** | | |
| --- | --- | --- | --- | --- | --- | --- | --- |
|  | **Factor 1: meat & eggs** | **Factor 2：fish, shrimp & soybeans** | **Factor 3: cereals &**  **potatoes** |  | **Factor 1: fruit, meat, fish & shrimp** | **Factor 2: eggs & dairy** | **Factor 3: cereals & potatoes** |
| % variance explained | 22.1% | 15.6% | 14.0% |  | 30.1% | 14.5% | 13.2% |
| Cereals/potatoes^a^ | 0.062 | –0.367 | 0.623 |  | 0.238 | 0.289 | 0.808 |
| Vegetables^a^ | 0.418 | –0.013 | 0.495 |  | 0.381 | -0.208 | 0.360 |
| Fruit^a^ | 0.479 | 0.253 | 0.399 |  | 0.791 | -0.071 | -0.102 |
| Livestock/poultry meat^a^ | 0.655 | –0.108 | –0.048 |  | 0.767 | 0.019 | 0.116 |
| Fish/shrimp^a^ | 0.120 | 0.678 | 0.100 |  | 0.788 | 0.124 | -0.149 |
| Eggs^a^ | 0.773 | –0.238 | –0.161 |  | 0.303 | 0.649 | -0.436 |
| Soybeans^a^ | 0.391 | 0.609 | –0.260 |  | 0.465 | -0.105 | -0.103 |
| Dairy^b^ | 0.407 | –0.386 | –0.468 |  | -0.254 | 0.760 | 0.161 |

^a^ g/day. ^b^ ml/day. The dietary patterns were named with factor loading values > + 0.60. ASD: autism spectrum disorder, TD: typically developing

**Supplementary Table 11** Comparisons of gastrointestinal symptoms between children with ASD and age-matched TD children (*n* = 242)

| **Gastrointestinal symptoms** | **ASD (*n* = 121)** | **TD (*n* = 121)** | ***p*** | **Effect size** |
| --- | --- | --- | --- | --- |
| Total scores, median (IQR) | 1 (0, 2) | 0 (0, 1) | <0.001 | 0.224 |
| Constipation, median (IQR) | 0 (0, 1) | 0 (0, 0) | 0.001 | 0.223 |
| Diarrhea, median (IQR) | 0 (0, 0) | 0 (0, 0) | 0.055 | 0.123 |
| Unformed stools, median (IQR) | 0 (0, 0) | 0 (0, 0) | 0.776 | 0.018 |
| Abnormal smell of stool, median (IQR) | 0 (0, 1) | 0 (0, 0) | 0.046 | 0.129 |
| Flatulence, median (IQR) | 0 (0, 0) | 0 (0, 0) | 0.104 | 0.105 |
| Abdominal pain, median (IQR) | 0 (0, 0) | 0 (0, 0) | 1.000 | 0.000 |

Gastrointestinal symptoms were evaluated by subscale and total scores of the 6-item gastrointestinal severity index (6-GSI). Effect size= Z/√(N) for Mann–Whitney U-tests. ASD: autism spectrum disorder, TD: typically developing, IQR: [interquartile range](javascript:;)

**Supplementary Table 12** Comparisons of dietary intake and gastrointestinal symptoms between children with ASD and age-matched TD children based on propensity score matching (*n* = 116)

| **Dietary and gastrointestinal indicators** | **ASD (*n* = 58)** | **TD (*n* = 58)** | ***p*** | **Effect size** |
| --- | --- | --- | --- | --- |
| Dietary intake |  |  |  |  |
| Cereals/potatoes^a^, median (IQR) | 187.5 (150, 300) | 200 (143.8, 262.5) | 0.715 | 0.034 |
| Vegetables^a^, median (IQR) | 71.4 (27.7, 150) | 100 (83, 200) | 0.008 | 0.248 |
| Fruit^a^, median (IQR) | 53.6 (41.1, 100) | 73.2 (50, 144.4) | 0.041 | 0.189 |
| Livestock/poultry meat^a^, median (IQR) | 100 (50, 200) | 150 (55.4, 200) | 0.078 | 0.164 |
| Fish/shrimp^a^, median (IQR) | 14.3 (6.4, 28.6) | 21.4 (14.3, 42.9) | 0.053 | 0.179 |
| Eggs^a^, median (IQR) | 23.2 (13.4, 42.9) | 28.6 (21.4, 50) | 0.328 | 0.091 |
| Soybeans^a^, median (IQR) | 7.1 (0, 14.3) | 14.3 (0, 21.4) | 0.147 | 0.135 |
| Dairy^b^, median (IQR) | 207.1 (85.7, 364.3) | 250 (178.6, 357.1) | 0.172 | 0.127 |
| Food variety^c^, mean ± SD | –5 (–6, –3) | –4 (–5, –2.8) | 0.024 | 0.210 |
| Drinking water^b^, median (IQR) | 750 (500, 1062.5) | 750 (500, 1000) | 0.588 | 0.050 |
| HBS, mean ± SD | 7 (4, 13) | 6 (4, 11.3) | 0.815 | 0.022 |
| LBS, mean ± SD | –24 (–29.3, –20) | –15 (–24, –10.8) | <0.001 | 0.341 |
| DQD, mean ± SD | 32.5 (25, 38.3) | 26 (17.8, 31.3) | <0.001 | 0.342 |
| Gastrointestinal symptoms |  |  |  |  |
| Total scores, median (IQR) | 1 (0, 2) | 0 (0, 1) | 0.013 | 0.230 |
| Constipation, median (IQR) | 0 (0, 1) | 0 (0, 0) | 0.005 | 0.258 |
| Diarrhea, median (IQR) | 0 (0, 0) | 0 (0, 0) | 0.560 | 0.054 |
| Unformed stools, median (IQR) | 0 (0, 0) | 0 (0, 0) | 0.404 | 0.078 |
| Abnormal smell of stool, median (IQR) | 0 (0, 1) | 0 (0, 0) | 0.322 | 0.092 |
| Flatulence, median (IQR) | 0 (0, 0) | 0 (0, 0) | 0.172 | 0.127 |
| Abdominal pain, median (IQR) | 0 (0, 0) | 0 (0, 0) | 0.698 | 0.036 |

^a^ g/day. ^b^ ml/day. ^c^ Food variety scores ranged from –12 to 0, with lower scores representing less variety of food. Excessive/inadequate/unbalanced dietary intake was evaluated using the high bound score (HBS), low bound score (LBS), and diet quality distance (DQD), respectively. Gastrointestinal symptoms were evaluated by subscale and total scores of the 6-item gastrointestinal severity index (6-GSI). Effect size= Z/√(N) for Mann–Whitney U-tests. ASD: autism spectrum disorder, TD: typically developing, IQR: [interquartile range](javascript:;), SD: standard deviation

**Supplementary Table 13** Comparisons of restricted repetitive behaviors, sensory profiles, and mealtime behaviors between children with ASD and age-matched TD children (*n* = 242)

| **Autism-linked traits** | **ASD (*n* = 121)** | **TD (*n* = 121)** | ***p*** | **Effect size** |
| --- | --- | --- | --- | --- |
| RBS-R |  |  |  |  |
| Total scores, median (IQR) | 15 (8, 27) | NA | NA | NA |
| Stereotypic behavior, median (IQR) | 4 (1, 6) | NA | NA | NA |
| Self-injurious behavior, median (IQR) | 0 (0, 2) | NA | NA | NA |
| Compulsive behavior, median (IQR) | 2 (1, 5) | NA | NA | NA |
| Ritualistic behavior, median (IQR) | 3 (1, 5) | NA | NA | NA |
| Sameness behavior, median (IQR) | 3 (1, 7) | NA | NA | NA |
| Restricted interests, median (IQR) | 2 (0, 4) | NA | NA | NA |
| SSP |  |  |  |  |
| Total scores, median (IQR) | 139 (128, 152) | 158 (146, 169) | <0.001 | 0.461 |
| Taste/smell sensitivity, median (IQR) | 15 (12, 18) | 16 (14, 18) | 0.004 | 0.185 |
| Tactile sensitivity, median (IQR) | 31 (29, 33) | 32 (29, 34) | 0.012 | 0.162 |
| Movement sensitivity, median (IQR) | 13 (11, 15) | 14 (12, 15) | 0.001 | 0.210 |
| Underresponsive/seeks sensation,  median (IQR) | 23 (20, 27) | 28 (23, 30.5) | <0.001 | 0.324 |
| Auditory filtering, median (IQR) | 19 (16, 21) | 23 (20, 27) | <0.001 | 0.483 |
| Low energy/weak, median (IQR) | 20 (16, 23.5) | 26 (21, 29) | <0.001 | 0.494 |
| Visual/auditory sensitivity, median (IQR) | 21 (18, 23) | 22 (19.5, 24) | 0.027 | 0.142 |
| BAMBI |  |  |  |  |
| Total scores, median (IQR) | 39 (33, 44) | 35 (31, 40) | 0.002 | 0.203 |
| Food refusal, median (IQR) | 8 (6, 10) | 7 (5, 9) | 0.034 | 0.136 |
| Limited variety of foods, median (IQR) | 20 (16, 24) | 19 (15, 21) | 0.005 | 0.181 |
| Features of autism, median (IQR) | 10 (9, 12) | 10 (8, 11.5) | 0.063 | 0.119 |

Restricted repetitive behaviors, sensory profiles, and mealtime behaviors were evaluated by subscale and total scores of repetitive behavior scale-revised (RBS-R), short sensory profile (SSP), and brief autism mealtime behavior inventory (BAMBI), respectively. Effect size= Z/√(N) for Mann–Whitney U-tests. ASD: autism spectrum disorder, TD: typically developing, IQR: [interquartile range](javascript:;), NA: not applicable

**Supplementary Table 14** Children with ASD experienced more severe sensory abnormalities than age-matched TD children (*n* = 242)

| **Sensory profiles** | **Crude β coefficient (95% CI)** | ***p*** | **Effect size** | **Adjusted β coefficient (95% CI)** | ***p*** | **Effect size** |
| --- | --- | --- | --- | --- | --- | --- |
| Taste/smell sensitivity | –1.405 (–2.301 to –0.509) | 0.002 | 0.040 | –1.102 (–2.103 to –0.101) | 0.031 | 0.093 |
| Movement sensitivity | –1.025 (–1.551 to –0.499) | <0.001 | 0.062 | –1.023 (–1.701 to –0.345) | 0.003 | 0.146 |
| Underresponsive/seeks sensation | –3.174 (–4.404 to –1.943) | <0.001 | 0.107 | –2.702 (–4.295 to –1.110) | 0.001 | 0.186 |
| Auditory filtering | –4.579 (–5.640 to –3.517) | <0.001 | 0.300 | –4.035 (–5.413 to –2.657) | <0.001 | 0.383 |
| Low energy/weak | –5.223 (–6.398 to –4.049) | <0.001 | 0.319 | –5.306 (–6.804 to –3.809) | <0.001 | 0.456 |
| Visual/auditory sensitivity | –1.083 (–1.931 to –0.235) | 0.013 | 0.027 | –1.315 (–2.416 to –0.215) | 0.019 | 0.093 |
| Sensory symptoms | –17.628 (–22.044 to –13.212) | <0.001 | 0.258 | –16.191 (–21.828 to –10.554) | <0.001 | 0.385 |

Sensory profiles were evaluated by subscale and total scores of the Short Sensory Profile (SSP). Adjusted for child’s age, sex, intellectual functioning, birth mode, birth order, average daily sleep duration, maternal educational attainment, gestational diabetes mellitus, maternal obesity, and monthly per-capita income. Effect size= Cohen's f^2^ for linear models. ASD: autism spectrum disorder, TD: typically developing, CI: confidence interval

**Supplementary Table 15** Children with ASD had more severe mealtime behavior problems than age-matched TD children (*n* = 242)

| **Mealtime behavior problems** | **Crude β coefficient (95% CI)** | ***p*** | **Effect size** | **Adjusted β coefficient (95% CI)** | ***p*** | **Effect size** |
| --- | --- | --- | --- | --- | --- | --- |
| Limited variety | 1.967 (0.717 to 3.217) | 0.002 | 0.040 | 1.554 (0.167 to 2.941) | 0.028 | 0.045 |
| Mealtime behaviors | 3.347 (1.328 to 5.367) | 0.001 | 0.110 | 2.296 (0.151 to 4.441) | 0.036 | 0.284 |

Mealtime behaviors were evaluated by subscale and total scores of the Brief Autism Mealtime Behavior Inventory (BAMBI). Adjusted for child’s age, sex, intellectual functioning, birth mode, birth order, average daily sleep duration, maternal educational attainment, gestational diabetes mellitus, maternal obesity, and monthly per-capita income. Effect size = Cohen's f^2^ for linear models. ASD: autism spectrum disorder, TD: typically developing, CI: confidence interval

=
